# The cholesterol biosynthesis enzyme oxidosqualene cyclase is a new target to impair tumour angiogenesis and metastasis dissemination

**DOI:** 10.1038/srep09054

**Published:** 2015-03-12

**Authors:** Federica Maione, Simonetta Oliaro-Bosso, Claudia Meda, Federica Di Nicolantonio, Federico Bussolino, Gianni Balliano, Franca Viola, Enrico Giraudo

**Affiliations:** 1 Laboratory of Transgenic Mouse Models, Candiolo Cancer Institute – FPO, IRCCS, Str prov 142 Km 3.95. 10060, Candiolo, Italy; 2 Laboratory of Cancer Epigenetics, Candiolo Cancer Institute – FPO, IRCCS, Str prov 142 Km 3.95. 10060, Candiolo, Italy; 3 Laboratory of Vascular Oncology, Candiolo Cancer Institute, IRCCS, Str prov 142 Km 3.95. 10060, Candiolo, Italy; 4 Department of Oncology, University of Turin, 10100, Turin, Italy; 5 Laboratory of Biochemistry and Molecular Biology, University of Turin, Via P. Giuria 9, 10125, Turin, Italy; 6 Department of Science and Drug Technology, University of Turin, Via P. Giuria 9, 10125 Turin, Italy

## Abstract

Aberrant cholesterol homeostasis and biosynthesis has been observed in different tumour types. This paper investigates the role of the post-squalenic enzyme of cholesterol biosynthesis, oxidosqualene cyclase (OSC), in regulating tumour angiogenesis and metastasis dissemination in mouse models of cancer. We showed that Ro 48-8071, a selective inhibitor of OSC, reduced vascular density and increased pericyte coverage, with a consequent inhibition of tumour growth in a spontaneous mouse model of pancreatic tumour (RIP-Tag2) and two metastatic mouse models of human colon carcinoma (HCT116) and pancreatic adenocarcinoma (HPAF-II). Remarkably, the inhibition of OSC hampered metastasis formation in HCT116 and HPAF-II models. Ro 48-8071 induced tumour vessel normalization and enhanced the anti-tumoral and anti-metastatic effects of 5-fluorouracil (5-FU) in HCT116 mice. Ro 48-8071 exerted a strong anti-angiogenic activity by impairing endothelial cell adhesion and migration, and by blocking vessel formation in angiogenesis assays. OSC inhibition specifically interfered with the PI3K pathway. According to *in vitro* results, Ro 48-8071 specifically inhibited Akt phosphorylation in both cancer cells and tumour vasculature in all treated models. Thus, our results unveil a crucial role of OSC in the regulation of cancer progression and tumour angiogenesis, and indicate Ro 48-8071 as a potential novel anti-angiogenic and anti-metastatic drug.

The critical role of angiogenesis in regulating tumour growth and metastasis formation has long been appreciated^1^. Though several anti-angiogenic drugs, such as inhibitors of the vascular endothelial growth factor (VEGF) pathway, have been approved in clinical practice for the treatment of different tumor types, the results of clinical trials have not replicated the promising effects observed in preclinical models in terms of metastasis formation and progression^2^,^3^. Hence, there is a pressing need to identify new angiogenic targets and therapeutic strategies to improve the current anti-angiogenic treatments in cancer patients. Recent findings have highlighted a crucial role played by the metabolism in regulating angiogenesis in several diseases, and there is a growing interest in elucidating the underlying mechanisms in order to find new “metabolic” targets and drugs to inhibit angiogenesis in cancers^4^,^5^.

In the framework of the role of metabolism and tumours, it is widely known that aberrant regulation of cholesterol homeostasis has been reported to occur in multiple types of cancer^6^,^7^. Several inhibitors of the cholesterol pathway have been described to modulate both tumour growth and angiogenesis. For instance, statins, HMGCoA reductase (HMGR) inhibitors, displayed a biphasic effect both in promoting and inhibiting angiogenesis and tumour growth^8^. Side effects can be partly explained by the fact that the sterol biosynthesis pathway supplies the prenyl intermediates used for post-translational modifications of proteins. In turn, prenylation regulates intracellular localisation and the activity of several signalling transducers, such as small GTPases that are prominently involved in regulating cancer progression^7^. Terbinafine and itraconazole are two antifungal drugs that impair the post-squalenic steps of cholesterol synthesis. The anti-angiogenic properties of these drugs have been previuosly described, as they inhibit proliferation and differentiation of human endothelial cells (ECs)^9^,^10^. More recently, itraconazole has been shown to impair tumor growth and angiogenesis in primary xenograft models of human non-small cell lung cancer^11^ and in a mouse model of medulloblastoma^12^ and terbinafine has been shown to inhibit tumor growth and angiogenesis^13^. Terbinafine is an effective inhibitor of squalene monooxygenase^14^, while itraconazole blocks sterol biosynthesis by inhibiting 14-demethylase^15^ (Figure 1A). Both compounds inhibit the sterol biosynthetic pathway after the formation of prenyl intermediates, suggesting that the anti-angiogenic effect should be based on a mechanism other than inhibition of the mevalonate pathway and of isoprenoid biosynthesis, which has been suggested as a cause of the anti-angiogenic and anti-tumoral activity observed in statins^7^,^8^. Though these findings suggest an important role of post-squalenic enzymes and of their inhibitors in tumor growth and angiogenesis, to date none of the described compounds have been described to efficiently impair tumor angiogenesis in parallel with a significant inhibition of metastasis formation. Moreover, the mechanisms that regulate the relationship between tumour angiogenesis and cholesterol biosynthesis are widely unknown.

In the present work, we studied the role of an important post-squalenic enzyme, 2,3-oxidosqualene lanosterol cyclase (OSC), that cyclizes the 2,3-oxidosqualene leading the formation of lanosterol, a key intermediate in the biosynthesis of cholesterol (Figure 1A). We assessed whether blocking sterol synthesis with the selective OSC inhibitor Ro 48-8071, shown to significantly decrease sterol biosynthesis and cell cholesterol contents in both cell culture and animals^16^, could induce anti-angiogenic and anti-metastatic effects in preclinical cancer models. Similarly to terbinafine and itraconazole, Ro 48-8071 is able to significantly decrease the sterol biosynthesis and cell cholesterol contents, without affecting the formation of the prenyl intermediates^17^.

This paper describes the activity of Ro 48-8071 as a potent inhibitor of tumor-associated angiogenesis, cancer growth and metastasis dissemination to distal organs in a spontaneous mouse model of neuroendocrine pancreatic tumor (RIP-Tag2), in human colon carcinoma (HCT116) and in pancreatic ductal adenocarcinoma (HPAF-II) xenograft mouse models. We show improved anti-tumoral efficacy by combining Ro 48-8071 with standard cytotoxic chemotherapy. Inhibition of OSC specifically blocked the PI3K/Akt signalling pathway in both tumour vasculature and cancer cells. These studies are the first assessment of the efficacy of Ro 48-8071 as an anti-angiogenic and anti-metastatic agent in murine cancer models.

## Results

### The inhibition of OSC impairs tumor growth and blocks metastatic dissemination in RIP-Tag2, HCT116 and HPAF-II mouse models

**Figure 1:**
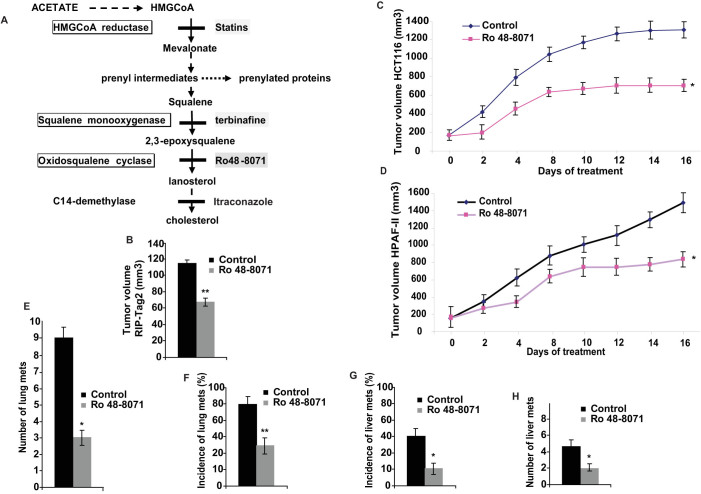
Ro 48-8071 inhibits primary tumour growth in a spontaneous mouse model of pancreatic cancer and blocks metastasis formation in both HCT116 and HPAF-II models. (A) Representation of cholesterol biosynthesis. This process includes a pre-squalenic phase and a post-squalenic step and offers a number of potential therapeutic targets, which can affect the mevalonate pathway (statins) or block, downstream of the formation of prenyl intermediates, the synthesis of lanosterol and cholesterol (e.g. OSC and C14-demethylase, inhibited by Ro 48-8071 and itraconazole, respectively). (B) 10 mg/kg of Ro 48-8071 or vehicle as control were orally administrated to established tumour-bearing mice (n = 10/group). Total tumor burden was reduced by 40% in RIP-Tag2 mice treated with Ro 48-8071 versus controls (n = 10; **P < 0.001). (C,D) Tumor growth curve of HCT116 and HPAF-II xenografts. Mean ±SEM tumor volumes are reported for each treatment of HCT116 (C) and HPAF-II (D) xenograft mouse models (n = 10; *P < 0.05). (E–G) Metastatic dissemination per animal. Treatment with Ro 48-8071 reduced the number (E) and the incidence (F) of lung metastasis in HCT116 mice by 81% and 53%, respectively, compared with controls, and decreased the incidence (G) and number (H) of liver metastasis by 75% and 60% respectively in HPAF-II mice (*P < 0.05; **P < 0.001).

The pharmacological properties of Ro 48-8071 as a selective inhibitor of the post-squalenic enzyme OSC have been well characterised, and Ro 48-8071 is known to block human liver OSC and cholesterol synthesis at nanomolar range^16^. First, we treated with Ro 48-8071 tumor-bearing RIP-Tag2 mice, a transgenic mouse model of spontaneous neuroendocrine pancreatic cancer. This is a well-characterised mouse model and suitable platform that is widely used to perform pre-clinical trials to assess drug ability to impair angiogenesis and inhibit tumor growth^18^,^19^. Based on several *in vivo* evidences^16^,^20^ and our preliminary results, we set up the most effective and less toxic concentration of Ro 48-8071 (data not shown). We found that RIP-Tag2 orally receiving 10 mg/kg/daily of Ro 48-8071 did not show a significant decrease in total body weight during the treatment, and healthy organs, such as kidney, lung and liver, were not affected (Supplementary Figures S1A–S1D). Therefore, we performed a regression trial of 4 weeks in RIP-Tag2 mice, from the 12^th^ until the 16^th^ week of age. This treatment is aimed at targeting advanced, well-established cancers and at testing the ability of a drug to shrink large tumours. Interestingly, while vehicle-treated RIP-Tag2 mice died at 14 weeks of age, all the Ro 48-8071 treated mice were still alive until the end of the regression trial (16 weeks) and displayed an inhibition of tumour growth by 40%, compared with untreated mice (Figure 1B). Next, we assessed whether OSC inhibition was able to block cancer progression in human cancer cells and, to this end, we employed two different xenograft mouse models of colon carcinoma (HCT116) and pancreatic ductal adenocarcinoma (HPAF-II). Of note, as observed in RIP-Tag2 mice, both HCT116 and HPAF-II models harbouring established tumors and treated with Ro 48-8071 for two weeks, showed a significant tumor volume reduction by 46% and 47%, respectively, compared with vehicle-treated controls (Figures 1C and D). Remarkably, Ro 48-8071 strongly reduced metastasis dissemination to distal organs. Ro 48-8071 decresed the number (81%) and the incidence (53%) of lung metastasis in HCT116 xenograft (Figures 1E and F), and the incidence (75%) and number (60%) of liver metastasis in HPAF-II mice, compared with controls. (Figures 1G and 1H).

### Ro 48-8071 strongly impairs tumour angiogenesis and normalizes vasculature in RIP-Tag2, HCT116 and HPAF-II mouse models

**Figure 2:**
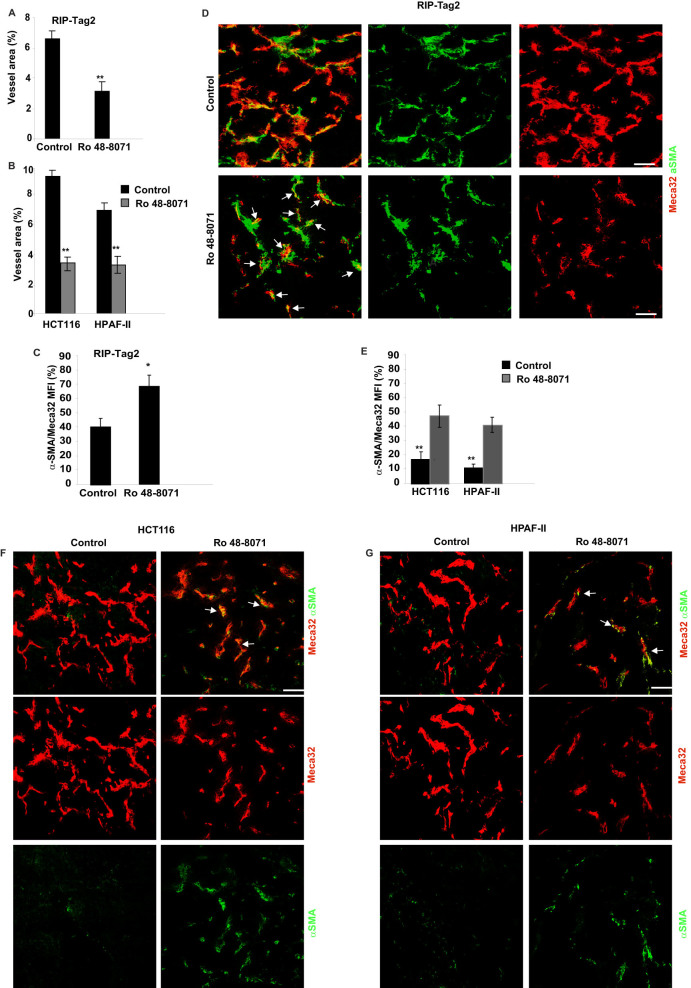
OSC inhibition reduces vessel area and increases pericyte coverage in RIP-Tag2 mice and in HCT116 and HPAF-II models. (A, B) The percentage of surface area occupied by vessels was quantified as Meca32 positive staining. Bars show vessel density reduction in RIP-Tag2 (A), and HCT116 and HPAF-II tumors (B) treated with Ro 48-8071, compared with controls, by 49%, 67% and 56%, respectively (**P < 0.01). (C) Pericyte coverage was quantified by means of co-localisation between Meca-32 and α SMA. This analysis revealed an increase in pericyte coverage of 44% in Ro 48-8071 treated RIP-Tag2, compared with untreated mice (**p < 0.01). (D) Fluorescence confocal microscopy highlighted an increase in pericyte coverage (green) of tumor blood vessels (red) after 4 weeks of Ro 48-8071 treatment of RIP-Tag2 mice, compared with controls (arrows). (E) The graph bar shows a 33% increase in pericyte coverage in HCT116, and a 31% increase in HPAF-II, compared with controls (**p < 0.01). (F, G) Representative images of co-localisation between the endothelial marker Meca32 (red) and the pericyte marker α SMA (green) in both HCT116 (E) and HPAF-II (F) tumors. Scale bar 50 μm.

OSC inhibition efficiently reduced vessel area in RIP-Tag2 mice by 49% (Figure 2A), and in both HCT116 and HPAF-II xenografts by 67% and 56%, respectively (Figure 2B). Interestingly, we observed a strong increase in pericyte coverage in tumor vessels of treated mice (by 44% in RIP-Tag2, by 33% in HCT116 and by 31% in HPAF-II), compared with controls (Figures 2C–G). Both results indicated that OSC inhibition in cancers strongly impairs angiogenesis and induces tumor blood vessel normalization, which, in turn, could contribute to the reduction of metastasis formation^21^,^22^. Remarkably, Ro 48-8071 delivery did not affect the normal vasculature of either exocrine pancreas (Supplementary Figures S2A and S2B) or normal islets (data not shown) in late-stage tumor bearing RIP-Tag2 mice, indicating that OSC inhibition results in a selective impairment of cancer angiogenesis. Notably, by means of Pimonidazole immunostaining we checked the hypoxic levels of RIP-Tag2 transgenic mice and of HCT116 and HPAF-II xenografts treated with Ro 48-8071 in comparison with the corresponding untreated mice. Interestingly, we found that Ro 48-8071 significantly reduced hypoxia across all treatment groups (Supplementary Figures S3A–S3D). This result further confirms that Ro 48-8071, by inducing the normalization of tumor blood vessels significantly improves the total tissue oxygenation.

### By improving tumor tissue perfusion, Ro 48-8071 enhances the anti-tumoral effect of 5- Fluorouracil in human colon carcinoma

**Figure 3:**
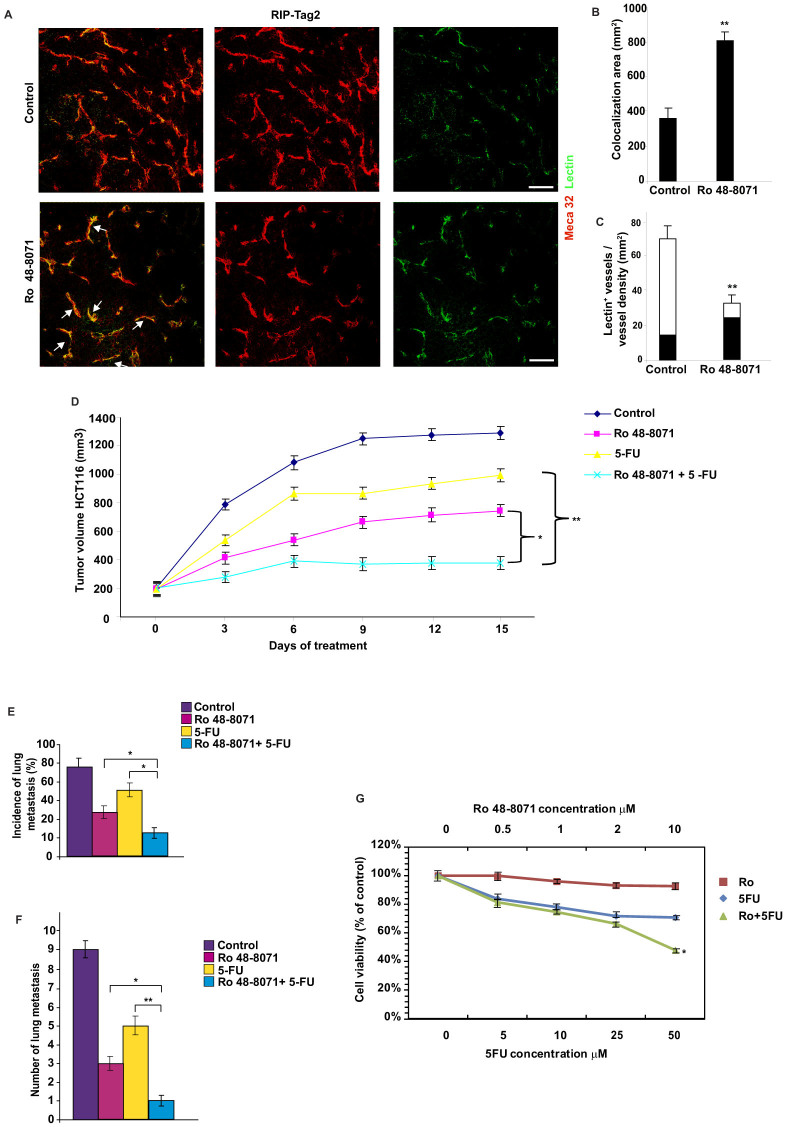
Ro 48-8071 improves tissue perfusion and enhances 5-Fluorouracil anti-tumoral effect in human colon carcinoma by reducing tumour volume and metastasis formation. (A) Ro 48-8071 increases the amount of FITC-lectin perfused vessels (arrows), compared with untreated insulinomas. Results are from 5 fields per mouse (n = 10 per treatment group). (B) Colocalization analysis between lectin perfused vessels (green) and Meca32 (red) in RIP-Tag2 mice. Quantification is shown as mean ± SEM of colocalization area (in mm^2^) *P < 0.05, Student t- test. (C) The graph shows in black the fraction of lectin-positive vessels normalized on total vessels (perfused vessel density) and in white the percentage of not-perfused vessels on the total vessel number. Ro 48-8071 increased by 50% the lectin-positive vessels on the total vessel number, compared with controls. (** P < 0.01, Student t- test.). (D) HCT116 mice were treated with: (1) 5-FU 30 mg/kg, (2) Ro 48-8071, (3) 5-FU + Ro 48-8071 or (4) vehicle for 2 weeks after tumour volume reached an average of 150-200 mm^3^ (n = 10/group). Mean ±SEM tumor volumes are reported for each treatment. (*P < 0.05 and *P < 0.01; ANOVA test.) Tumor growth plot showed that 5-FU or Ro48-8071 monotherapy resulted in 25% and 46% reduction, respectively, whereas combined Ro 48-8071 and 5-FU resulted in 71% cancer growth inhibition, compared with the vehicle-treatment group. (E,F) Combined Ro 48-8071 and 5-FU diminished both the incidence (E) and number (F) of lung metastases by 83% and 89%, respectively. (*P < 0.05 and *P < 0.01 ANOVA test). (G) The graph shows *in* *vitro* tumor cell proliferation measured as cell viability (% of control) upon Ro 48-8071, 5-FU and Ro 48-8071 + 5-FU administration. No statistical significant differences in cell proliferation were observed at concentrations up to 10 μM of Ro 48-807, while 5-FU + Ro 48-8071 co-administration shows an higher cytotoxic effect only at the top concentration of 50 μM 5-FU compared to 5-FU alone. (*P < 0.05 ANOVA test).

As previously demonstrated, the "vascular normalization" process is characterised by attenuation of abnormal tumor vessel features. Indeed, increased pericyte coverage and a more restrained vascular network are markers of restored tumor vessel function^21^,^22^,^23^. To further confirm the increase in tumor vessel normalization induced by Ro 48-8071, we analysed the effect of OSC inhibition on tumor vessel perfusion. Immunostaining of tumor sections from RIP-Tag2 mice revealed that Ro 48-8071 treatment greatly improved vessel function, as revealed by labelling perfused blood vessels with FITC-conjugated lectin injected into the circulation of tumor-bearing mice (Figure 3A and B). Moreover, perfused vessel density analysis revealed an increase by 50% of lectin-positive vessels on total vessel number in Ro 48-8071 treated RIP-Tag2 compared with controls (Figure 3C). Based on the observed increase in tumor vessel normalization induced by Ro 48-8071, we investigated whether this drug was able to enhance the anti-cancer effects of chemotherapy. It was interesting to observe that HCT116 xenografted mice treated with a combination of 5-fluorouracil (5-FU) (30 mg/kg, every 5 days) and Ro 48-8071 presented a greater effect in terms of impaired tumor progression. Indeed, 5-FU monotherapy resulted in a 25% reduction in tumor burden, compared with the vehicle-treatment group, whereas the addition of Ro 48-8071 to this regimen resulted in 71% cancer growth inhibition, compared to the control group (Figure 3D). The combination of 5-FU with Ro 48-8071 reduced the incidence (83%) and the number (89%) of lung metastasis more dramatically, compared to vehicle-treated animals, and compared to 5-FU administered as a single drug, which was less efficient in halting metastasis dissemination (incidence dropped by 33%, and the number of lung metastases by 44% versus controls) (Figures 3E and F). We next assessed whether results obtained *in vivo* with combination therapy could be attributed to a direct effect on tumour cell growth. To this end, we studied the effect of 5-FU, Ro 48-8071 and 5-FU + Ro 48-8071 on cell growth inhibition in cell lines of colorectal cancer (HCT116). As expected, 5-FU displayed a decrease in tumor cell proliferation, as assessed by cell viability assay, compared with untreated cells. On the other hand, Ro 48-8071 did not significantly induce growth inhibition at concentrations up to 10 μM. Notably, 5-FU + Ro 48-8071 co-administration in HCT116 cells led to a slightly higher cytotoxic effect, compared to 5-FU alone only at the top concentration of 50 μM 5-FU (Figure 3G). However, this exceeds the range of plasma concentrations measured in colorectal cancer patients receiving infusion 5-FU^24^. Therefore, we believe that the anti-tumoral effect of Ro 48-8071 *in vivo* could be mainly attributed to tumor blood vessel normalization, which enhances the delivery efficiency of chemotherapeutic drugs within the neoplastic mass.

### Ro 48-8071 suppresses tumor proliferation and increases apoptosis both in vessels and cancer cells in RIP-Tag2 mice and in HCT116 and HPAF-II models

**Figure 4:**
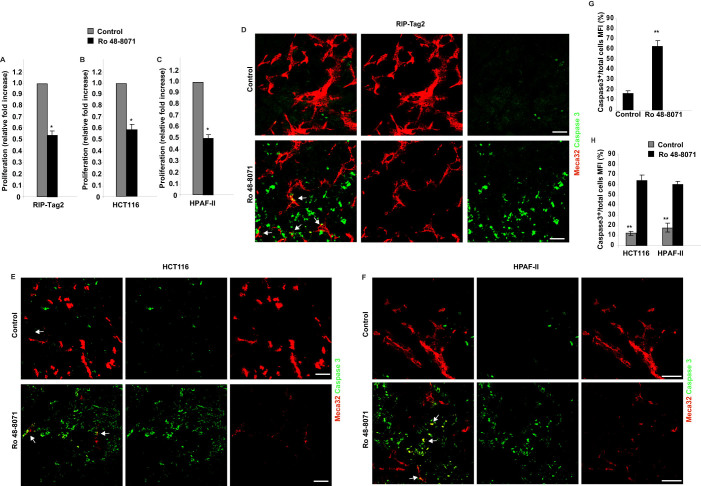
Ro 48-8071 hampers tumor proliferation and increases apoptosis both in vessels and cancer cells. (A–C) Quantification of proliferating cells by Ki-67 staining in RIP-Tag2 (A), HCT116 (B) and HPAF-II (C) tumours. Bars show the mean of Ki-67 positive cells/total cells in a 10X field ±SD (*P < 0.05). (D–F) Sections were immunostained for Meca32 (red) and cleaved-caspase 3 (green). Ro 48-7180 treated RIP-Tag2 (D), HCT116 (E) and HPAF-II (F) tumors displayed an increase in apoptotic rate in both tumor and endothelial cells (arrows), compared with controls. White arrows point to apoptotic vessels. Images are representative of 5 fields per mouse. (G,H) The graph bars display an increase in tumor cell apoptosis in RIP-Tag2 (by 73%) and in both HCT116 and HPAF-II xenografts models (by 79% and 68%, respectively), as compared to vehicle-treated controls (**p < 0.01). Scale bar 50 μm.

OSC inhibition by Ro 48-8071 reduced proliferation levels by 51% in RIP-Tag2 mice and by 58% and 52% in HCT116 and HPAF-II xenografts, respectively (Figures 4A–C; Supplementary Figures S4A–S4C). Concomitantly, Ro 48-8071 exerted a strong pro-apoptotic effect on the different models (Figures 4D–H). Remarkably Ro 48-8071 induced tumor vessel apoptosis in both RIP-Tag2 and HCT116 and HPAF-II mice (Figures 4D–F). Of note, *in vitro* assays clearly showed that Ro 48-8071 was unable to directly inhibit cell proliferation in both tumour cell lines, unless a very high concentration of 30 μM was applied (Supplementary Figures S5A and S5B), which may reflect the lack of specificity and off-target effects of Ro 48-8071 at supraphysiological doses. Together these findings suggest that the anti-tumoral effect obtained by OSC inhibition is mainly mediated by the strong anti-angiogenic effect of Ro 48-8071, although we cannot rule out a direct cytotoxic effect on cancer cells when the compound is used at high doses exceeding 10 μM.

### Inhibition of OSC *in vitro* impairs EC migration and adhesion, and blocks vessel network formation in Matrigel and CAM assays

**Figure 5:**
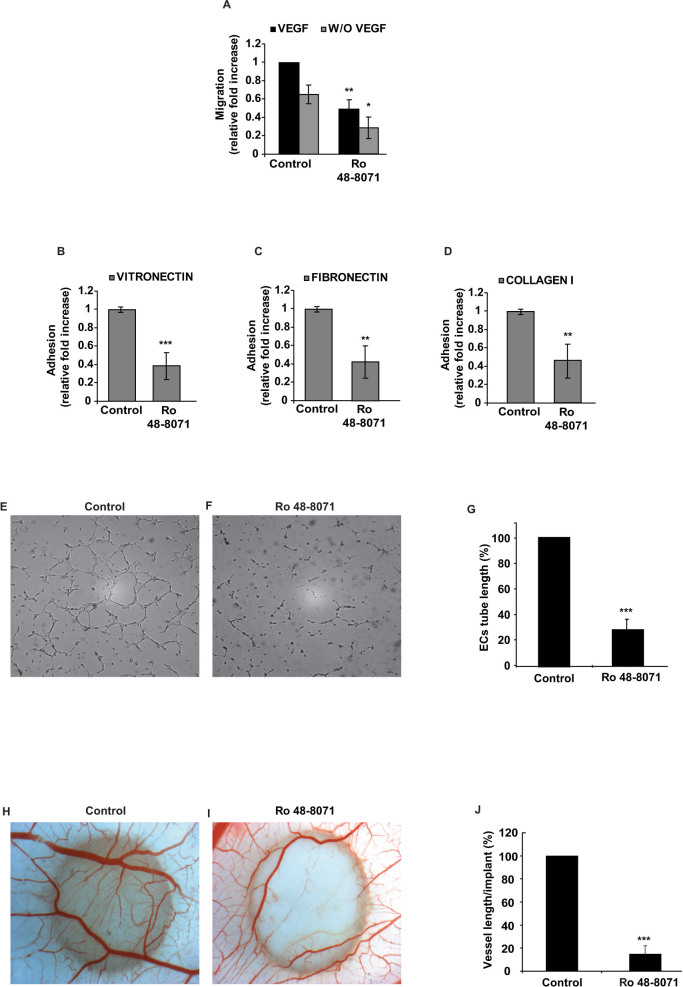
Ro 48-8071 impairs EC migration, adhesion and blocks vessel formation in Matrigel and CAM assays. (A) Chemotaxis assay, using ECs treated with Ro 48-8071 (1 μM), revealed a significant decrease in both baseline and VEGF-induced EC migration (* p < 0.05; ** p < 0.01). Values are mean ±SD (n = 3 filter/condition) of 6 independent experiments. (B, C, D). EC adhesion to vitronectin (B), fibronectin (C) and collagen I (D) matrices in the presence of Ro 48-8071 (1 μM), (***p < 0.001; **p < 0.01). Values are mean ±SD (n = 4 wells/condition) of 5 independent experiments. (E–G) EC morphogenesis was evaluated by Matrigel assay. The tubular vessel network formation was significantly impaired by Ro 48-8071 (***p < 0.001), compared to controls. Values are mean ±SD. EC tube length was measured by the software winRHIZO Pro (Regent Instruments Inc). Images are representative of 3 independent experiments. (H–J) CAM assay was employed to analyse the effect of sterol inhibitors on *in vivo* angiogenesis. R 48-8071 strongly inhibited FGF-2-induced vessel formation by 84% (I), compared to FGF-2-treated controls. (H) The bar graphs show the number of vessels per implant (J). Data are presented as mean ±SD of 18 embryos per treatment. (**p < 0.01; ***p < 0.001)

Based on the strong observed effect of Ro 48-8071 on tumor angiogenesis, we sought to investigate the specific influence of this compound on endothelial cells. As known, the angiogenic process involves complex mechanisms, such as migration and adhesion of vascular cells to different extracellular matrix proteins^25^. Therefore, we first evaluated the ability of Ro 48-8071 to inhibit EC migration in a chemotaxis assay. We found that 1 μM of Ro 48-8071 was the most efficient concentration capable of inhibiting EC migration (data not shown). In fact, at this dosage, Ro 48-8071 significantly impaired both VEGF-induced and baseline EC migration by 52% and 57%, respectively, compared with the control group (Figure 5A). Moreover, Ro 48-8071 significantly inhibited the adhesion of ECs to different extracellular matrices, such as vitronectin (61%), fibronectin (58%) and collagen I (52%) (Figures 5B–D). Of note, 1 μM of Ro 48-8071 did not affect EC proliferation (Supplementary Figure S5C). In order to further confirm that Ro 48-8071 specifically interferes with EC motility, we performed a cell migration and invasion assays by employing both HCT116 and HPAF-II tumour cell lines. Notably, Ro 48-8071 did not inhibit cancer cells invasion in trans-well chamber inserts coated with Matrigel (Supplementary Figure S6). These results strengthen and corroborate our findings indicating that the inhibitory effect of Ro 48-8071 mainly acts by impairing EC motility and does not affect the proinvasive and prometastatic activity of both HCT116 and HPAF-II cells.

We used a Matrigel assay to evaluate whether OSC inhibition could impair vessel network formation. To this end, ECs were previously treated with 1 μM of Ro 48-8071 and re-suspended in starving medium supplemented with VEGF-A and seeded on Matrigel-coated plates. ECs treated with VEGF-A formed a proper vessel network, whereas Ro 48-8071 pretreatment significantly suppressed (70%) tube formation, compared with untreated controls. (Figures 5E–G)

Further, we checked the effects of the inhibitor in impairing *in vivo* angiogenesis by performing a CAM assay. Paper disks loaded with FGF-2 and Ro 48-8071 were added to the CAM at day 10 of development when vessel network is formed with a regular capillary plexus. Similarly to what we observed with the Matrigel assay, OSC inhibition impaired the angiogenic effect triggered by FGF-2 by 84%. (Figures 5H–J).

Itraconazole, has been found to efficiently inhibit chemotactic migration and HUVEC tube formation^26^. Indeed, comparing the anti-angiogenic effect of the two inhibitors, Ro 48-8071 was the most effective in inhibiting vessel network formation on Matrigel and CAM experimental models (Supplementary Figures S7A and S7B).

### OSC inhibition impairs PI3K/Akt pathway activation in both ECs and tumor cells

**Figure 6:**
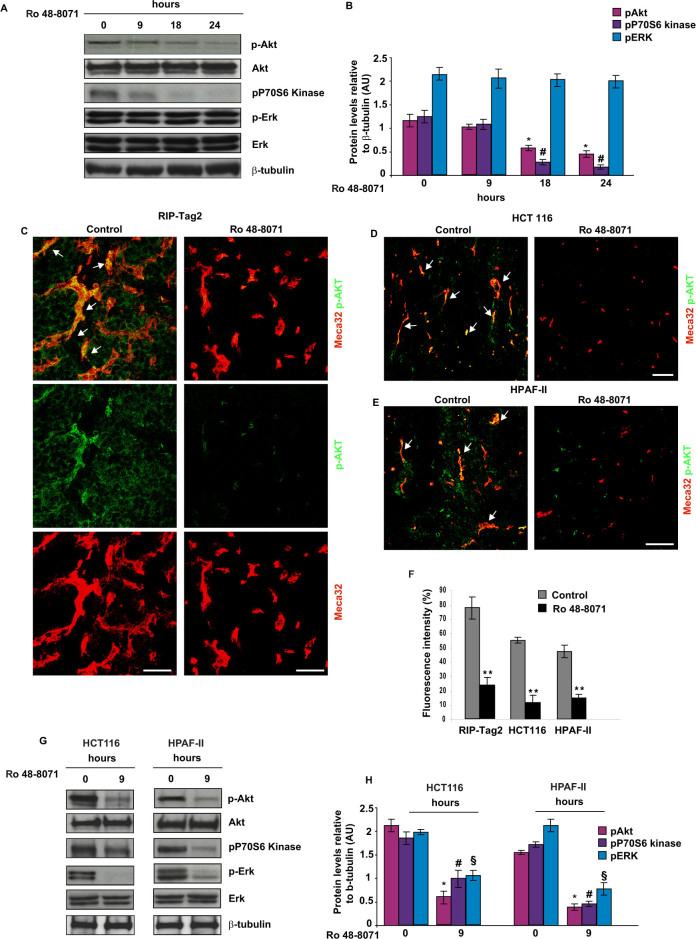
OSC inhibition blocks *in vitro* and *in vivo* PI3K/Akt pathway activation in endothelial and cancer cells. (A, B) Akt, ERK and P70S6K protein phosphorylation levels were evaluated by Western blot analysis of lysates from ECs upon treatment with Ro 48-8071 (1 μM) for 9, 18 and 24 h. (A) OSC inhibition was effective in decreasing Akt and P70S6K phosphorylation starting from 18 hours after incubation with Ro 48-8071, while p-ERK was not affected. (B) Relative protein levels of p-Akt and p-ERK were compared to total Akt and ERK, respectively, and normalized to β-tubulin. Values are mean ±SD (*p < 0.05 for Akt, for ^#^p < 0.05 pP70S6 kinase; ANOVA test). Western blot analysis of β-tubulin was used as loading control. The images shown are representative of 5 independent experiments. (C,E) Tumors from RIP-Tag2 (C) and HCT116 (D) and HPAF-II (E) xenografts treated with Ro 48-8071 and vehicle were subjected to confocal analysis, and Akt activation and localisation were assessed by co-staining of anti p-Akt with Meca32. Activated Akt was highly expressed in both vessels (arrows) and tumor cells in baseline conditions, and it was strongly inhibited after Ro 48-8071 treatment. The image presents confocal microscopy of 5 fields per mouse. (F) Akt activation level was quantified by fluorescence intensity mean (MFI) (**P < 0.01 with the Student T-test). (G, H) Western blot analysis on HCT116 and HPAF-II cancer cells showed an inhibition of Akt/P70S6K pathway and of ERK phosphorylation after 9 hours of incubation with Ro 48-8071(1 μM). Relative protein levels were normalised to β-tubulin. Values are mean ±SD, (*p < 0.05 for Akt, ^#^p < 0.05 for pP70S6 kinase, ^§^p < 0.05 for ERK; ANOVA test).

Based on the observed *in vivo* and *in vitro* anti-angiogenic effect of Ro 48-8071, we sought to investigate the molecular mechanisms by which it can affect EC motility and vessel formation. First, we evaluated the effect on Rho GTPases, since these proteins have previously been shown to be involved in cytoskeleton regulation and to control both physiological and pathological angiogenesis^27^,^28^. We did not detect any significant reduction in RhoA activity in ECs treated with Ro 48-8071 (Supplementary Figure S8). Next, we assessed whether Ro 48-8071 was able to impair PI3K/Akt and ERK signal pathways on ECs, a pivotal node in signalling events leading to angiogenesis^29^,^30^. Indeed, while OSC inhibition did not affect ERK phosphorylation, EC treatment with Ro 48-8071 for 18 and 24 hours strongly impaired Akt phosphorylation by 53% and 62%, respectively (Figures 6A and B).

To further confirm the inhibitory effect of Ro 48-8071 on mTOR/Akt pathway, we checked the activation level of ribosomal P70S6 kinase, which is located downstream of Akt and is phosphorylated in response to stimuli that activate the PI3K/Akt pathway. As observed for Akt, we found that P70S6K phosphorylation was significantly impaired (Figures 6A and B).

Based on these observations, we analysed whether Ro 48-8071 administration decreased Akt phosphorylation in tumors of the different treatment groups. As assessed by phospho-Akt immunostaining, we detected a dramatic decrease by 70%, 78% and 62%, respectively, in p-Akt levels in RIP-Tag2 and colon and pancreatic tumor xenografts, compared with vehicle-treated animals (Figures 6C–F). We observed a reduction in Akt activation in both cancer cells and tumor blood vessels in all cancer models (Figures 6C–E). Hence, we investigated whether *in vitro* inhibition of OSC could impair Akt activation also in HCT116 and HPAF-II cells. We found that Ro 48-8071 significantly inhibited Akt phosphorylation after 9 hours both in HCT116 and HPAF-II cells (Figures 6G and H). In addition, Ro 48-8071 inhibited ERK phosphorylation both in HCT116 and HPAF-II. These data indicate that Akt pathway inhibition could be one of the major signalling mechanisms by which Ro 48-8071 could exert its powerful anti-tumoral and anti-angiogenic effects.

## Discussion

Recent studies have reported that inhibitors of the post-squalenic steps of cholesterol synthesis, such as itraconazole, impaired angiogenesis and tumour growth^9^,^10^,^11^,^26^,^31^,^32^. However, their effect on vessel normalization and on metastasis has not been proven as yet. Notably, OSC, a key enzyme in the post-squalenic steps of sterol biosynthesis, could also be considered a potential therapeutic target for the control of cell proliferation and differentiation. For instance, OSC was significantly up-regulated in self-renewing cells (*i.e.* erythroid cells)^33^. Moreover, OSC inhibition was shown to significantly hamper the viability of different cancer cell lines at micromolar concentrations, comparable to those at which statins display an anti-tumoral effect^34^,^35^. It has been recently proven that specific OSC impairment can reduce the growth of oestrogen-dependent breast cancer^36^. Based on these data, the selective OSC inhibitor Ro 48-8071 is a potential and new anti-cancer drug. Our study demonstrates for the first time that Ro 48-8071 efficiently impaired experimental angiogenesis in several *in vitro* and *ex-vivo* assays, with stronger efficacy than the anti-angiogenic activity described for the other post-squalenic drug, itraconazole. Notably the inhibition of OSC strongly impaired angiogenesis, induced tumour vessel normalization, reduced tumor hypoxia and hampered tumor progression in a transgenic mouse model of neuroendocrine pancreatic tumor RIP-Tag2, and in two mouse models of human colon and pancreatic cancer. Differently from itraconazole and other postsqualenic drugs exerting anti-angiogenic effects, Ro 48-8071 strongly blocked metastatic dissemination to distal organs in both HCT116 and HPAF-II mouse models. In addition, we demonstrated that, differently from the other inhibitors of cholesterol biosynthesis, the treatment with Ro 48-8071, normalizing the vasculature, improved tumor vessel perfusion.

It is well known that tumor vessel normalization, a process that occurs in response to certain anti-angiogenic therapies, which enhance tumor vasculature efficiency in delivering oxygen and drugs, is a remarkably advantageous anti-cancer strategy, as it can also favour chemotherapy delivery and response to radiotherapy^21^,^22^,^37^. Interestingly, we noticed enhanced anti-tumoral and anti-metastatic effects when we combined Ro 48-8071 with the chemotherapeutic drug 5-FU in the HCT116 colon cancer model, compared to single treatments. Remarkably, the combination of Ro 48-8071 with 5-FU did not exert a direct effect on tumor cells, since neither additive nor synergistic effects on the proliferation rate of HCT116 cells were observed *in vitro* (and HPAF-II, data not shown), suggesting a specific effect of this drug on the tumor microenvironment. It is well demonstrated that increased tumor hypoxia promotes the activation of several hypoxia-driven phenomena, including the induction of cancer invasion and metastasis dissemination^38^,^39^. As described by Jain and colleagues and by other laboratories, the ameliorated blood vessel function induced by the treatment with pro-normalizing agents is able to improve cancer tissue oxygenation, promoting a less aggressive and metastatic tumor phenotype^40^,^41^. Of note, the observed decrease of cancer hypoxia in tumors treated with Ro 48-8071, suggests that the increase of tumor tissue oxygenation induced by vessel normalization, can be part of the anti-metastatic effect exerted by this inhibitor.

Based on the demonstrated correlation between the increase of cholesterol and triglycerides and cancer^6^, the use of this cholesterol-lowering agent against different tumor types in clinical practice may have a further advantage. A correlation has been described between hyperlipidaemia/hypercholesterolemia and progression of colon and pancreatic cancers^42^,^43^. Several clinical studies have employed statins to treat pancreatic cancer, considering their capacity to decrease blood cholesterol levels and reduce tumor cell proliferation. However, data on the anti-tumorigenic properties of statins in pancreatic cancer are still questionable, and information is scarce about the effect of statins in specific high-risk subgroups^44^. Hence, our data suggest that the treatment of these two tumor types with Ro 48-8071, in parallel to its anti-angiogenic effect, could help to reduce the elevated levels of circulating cholesterol and triglycerides in cancer patients, thus alleviating dyslipidaemia-induced tumor growth and metastasis.

It has been established that elevated cholesterol levels can lead to PI3K/Akt phosphorylation, and that cholesterol depletion on the membrane surface can inhibit PI3K/Akt and ERK pathways in several tumors^45^. mTOR/Akt inhibition, along with ERK 1/2 impairment, has been observed in ECs treated with itraconazole^26^,^32^. Similarly, Ro 48-8071 specifically inhibited Akt phosphorylation *in vitro* and *in vivo* both in tumor vessels and cancer cells across all models. The mTOR/Akt pathway is known to play a critical role in the regulation of several processes that control cell growth and proliferation^46^, as well as tumor progression and angiogenesis^30^,^47^. Our data suggest that part of the mechanisms by which Ro 48-8071 impairs angiogenesis and, importantly, normalizes tumour vasculature in pancreatic and colon cancers may be due to the impairment of Akt phosphorylation, suggesting that the specific inhibition of PI3K/Akt may represent a new strategy to normalize the tumor vasculature.

Although Ro 48-8071 reduced tumor growth and metastasis formation mainly by acting on tumor vasculature, we observed an inhibition of Akt phosphorylation also in tumor cell lines, suggesting that, at least in part, Ro 48-8071 can exert a direct effect on tumor cells.

Other mechanisms could be suggested to explain the specific effect on tumor progression, angiogenesis and vessel normalization exerted by Ro 48-8071, compared to other inhibitors of the post-squalenic step of cholesterol biosynthesis. It is known that cholesterol and its derivatives can regulate Hedgehog (Hh) synthesis and modulate Hh signalling^48^. Recent studies have demonstrated that itraconazole inhibited the Hh pathway in ECs^26^ and in tumour cells^12^. Notably, it has been shown that Sonic Hedgehog (Shh) induced capillary morphogenesis in ECs and activated bone marrow-derived EC progenitors by activating the PI3-kinase/Akt signalling pathway^49^,^50^. On the other hand, the Hh pathway is highly activated in many tumor types, including pancreatic and colon cancers^51^,^52^. Interestingly, it has been shown that oxysterols (OHCs), oxygenated derivatives of cholesterol, can enhance the activity of Smoothened (SMO), a member of Hh pathway, and contribute to tumour progression^48^. We can estimate that OSC inhibition by Ro 48-8071 in tumor vasculature and cancer cells may reduce OHC levels and, consequently, inhibit Hh and PI3-kinase/Akt pathways, thus impairing tumour angiogenesis and metastasis formation. Further studies are needed to better assess the mechanisms by which Ro 48-8071 directly impairs Akt signalling pathways or modulates other related pathways in cancers.

In conclusion, we unveiled the post-squalenic enzyme OSC as a crucial target to inhibit tumor angiogenesis, halt metastatic dissemination, normalize the vasculature and, consequently, enhance the efficacy of chemotherapeutic drugs, assigning to Ro 48-8071, its specific inhibitor, the role of potential novel anti-angiogenic and anti-metastatic drug.

## Methods

### Chemistry

Ro 48-8071 (Sigma-Aldrich) was dissolved in ethanol according to the manufacturer's instructions.

### Cell culture

Human umbilical vein ECs (HUVECs) were isolated from umbilical cord veins and grown as previously described^53^. Pancreatic ductal adenocarcinoma HPAF-II (CRL-1997) and colon carcinoma HCT116 (CCL-247) were purchased from American Type Culture Collection (ATCC, VA, USA) and grown according to the manufacture's instruction. Cells were routinely screened for the absence of mycoplasma contamination with the VenoräGeM Mycoplasma Detection Kit (Sigma Aldrich).

### Mouse tumor models

The RIP-Tag2 transgenic mouse model has been previously described^19^. Xenograft mice were generated by subcutaneously injecting either HPAF-II cells (5 × 10^5^ cells/mouse) into the right posterior flanks of 7-wk-old immunodeficient NOD/SCID male mice^54^, or HCT116 cells (2 × 10^5^ cells/mouse) into 7-wk-old immunodeficient NOD/SCID gamma (NSG) male mice (Charles River, MA, USA)^55^. Tumor growth was monitored twice a week. When tumors reached a volume of approximately 150–200 mm^3^, mice were randomised into the different treatment groups.

All animal procedures were approved by the Ethics Committee of the University of Turin, and by the Italian Ministry of Health, in compliance with international laws and policies.

### Therapeutic treatments

Tumor-bearing RIP-Tag2 mice (10/group) were treated for 4 weeks (from 12 to 16 weeks of age) by daily gavage of 10 mg/kg of Ro 48-8071 or vehicle (1% methylcellulose) as control group. Mice were regularly monitored for changes in weight and health status. Conversely, HCT116 or HPAF-II mice (10/group) were daily treated for two weeks. 5-fluorouracil alone or in combination with Ro 48-8071 was administered by intraperitoneal injection (30 mg/kg, every 5 days) to HCT116 xenograft mice. Total tumor burden was quantified by measuring with a caliper and estimating the volume of individually excised macroscopic tumours (>1 mm^3^) with the formula: V = a × b^2^ × 0.52, where a and b represent the longer and shorter diameter of the tumor, respectively.

### Metastasis analysis

In HCT116 xenografts the formation of superficial pulmonary metastasis in the lungs was analysed after two weeks of treatment by contrasting them with black India ink infusion, and counted on dissected lung lobes under a stereomicroscope^56^. The presence of liver metastasis in HPAF-II mice was assessed by analysing serial H&E-stained sections of paraffin-embedded tissues, as previously described^23^.

### Tissue preparation and immunohistochemistry analysis

Tissue preparation and histology analysis were carried out as previously described^23^. Immunohistochemistry and immunofluorescence analyses were performed as previously detailed^23^ with the following primary antibodies: purified Rat monoclonal anti-Panendothelial Cell antigen (550563, clone Meca32, BD Pharmingen, USA), diluted 1:100; Rabbit polyclonal anti-SMA (AB5694, Abcam, UK), diluted 1:100; Rabbit monoclonal anti-cleaved caspase 3 (asp175, clone 5A1, Cell Signaling, USA), diluted 1:100; Rabbit monoclonal anti-pAkt (S473, 4060 L, Cell Signaling) diluted 1:50; Rabbit polyclonal anti-Ki67 (AB15580, Abcam), diluted 1:100.

### Confocal microscopy quantifications

The surface area occupied by vessels was quantified through the Image-ProPlus 6.2 software (Media Cybernetics) as the area occupied by Meca32-positive structures, compared with the total tissue area visualised by DAPI. To quantify pericyte coverage (α-SMA, green channel) in each image, we drew a region of interest (ROI) close to each blood vessel (Meca32, red channel), and then quantified the mean fluorescence intensity (MFI) of red and green channels using the Leica Confocal Software Histogram Quantification Tool. In order to normalize the vessel number values obtained, we calculated the ratio between red and green channel MFI; values are expressed as percentage of red-green co-staining. To determine the expression levels of caspase 3 (green channel), and phospho-AKT (green channel) in each analysed image, we considered 5 random ROIs of the same size. Then we measured the MFI of the green channel, and we normalized the values by comparing caspase 3- or p-AKT- stained area with the total cells present in the tissue area^23^.

### Tumour vessel perfusion

To evaluate tumor vessel perfusion, 0.05 mg FITC-labelled tomato lectin (Vector laboratories, CA, USA) were injected i.v. into RIP-Tag2 mice, as previously described^23^. After 10 minutes, the animals were euthanised, and lectin distribution in co-staining with the endothelial marker Meca32 was visualised by fluorescent confocal microscopy. Tumor perfusion was quantified as colocalization signal between fluorescent lectin (red channel) and tumor blood vessels (green chennel) by means of ImageJ software maintaining the same area and fluorescent setting. At least 5 images at a magnification of 40× were analyzed for each sample, considering 8 mice per treatment group.

### Cell Viability Assay

2 × 10^3^ colon cancer cells (HCT116) were suspended in complete medium (RPMI-1640, 10% FCS) and seeded into 96-well plates. The next day drugs were added to triplicate wells at increasing concentrations corresponding to: 0.5 - 1 - 2 and 10 μM of Ro48-8071, 5 - 10 - 25 - 50 μM 5-FU or a combination of both at each dose. A control with no drug and consisting of media only was included in each plate. Incubation was stopped after 72 hours and cell viability following incubation was evaluated by CellTiter-Glo® Luminescent Cell Viability Assay (Promega, WI, USA) according to the manufacturer's recommendations using the VICTOR X Multilabel Plate Readers (Perkin Elmer, MA, USA). Values were normalized on controls and on the maximum doses of 5-FU, which killed all cells present. The percentage of cell viability/controls was evaluated. The quantification of the synergistic effect in these drug combination and dose-response studies was assessed by means of the Chou-Talalay method^57^, while an ANOVA test was used to determine the significance between the different experimental conditions.

### Migration assay

ECs (10,000 cells/well), pre-treated overnight with 1 μM of Ro 48-8071, were seeded on the upper surface of a polycarbonate 8-μm porous Transwell membrane (BD Falcon, USA) in the presence or absence of 10 ng/mL VEGF-A or 1 μM Ro 48-8071, while M199 serum free medium ± VEGF-A was added into the multiwell plate. Cells were allowed to migrate through the membrane for 4 h, fixed in 2.5% glutaraldehyde and stained with 0.1% crystal violet^58^.

### Cell adhesion assay

HUVECs were incubated with 1 μM of Ro 48-8071 overnight. The next day, 10^4^ ECs suspended in serum-free medium and supplemented with 1 μM Ro 48-8071 were added to the 96-well microtitre plate coated with 1 μg/mL or 1.5 μg/mL of rat collagen, human fibronectin and human vitronectin (Sigma Aldrich). After 30 minutes, cells were fixed and stained as previously described^51^.

### Matrigel assay

8 mg/mL Matrigel (BD Biosciences, USA) was added to each well of a 24-well plate and incubated at 37°C for 20 minutes to allow gel formation. Then 2 × 10^4^ cells suspended in serum free medium, VEGF-A (10 ng/mL) and 1 μM of Ro 48-8071 were plated. 12 hours after, capillary-like structures were photographed by the use of Image ProPlus program and quantified by winRHIZO Pro software (Regent Instruments Inc)^59^. Values were expressed as cell morphogenesis index obtained by normalizing the length of the tubular network to controls.

### Chick Chorioallantoic Membrane (CAM) assay

Fertilised chicken eggs were incubated at 37°C in a humidified incubator, as previously described^60^. On day 10 of incubation, CAM were added with sterilised paper disks treated with hydrocortisone (3 mg/mL). The disks were loaded with fibroblast growth factor (FGF-2, 100 ng/μL) in the presence or not of 1 μM Ro 48-8071. After 48 h of incubation, CAM vessels were isolated, fixed and photographed *in ovo* with a stereomicroscope using the Image ProPlus analysis software. Angiogenesis was measured as number of vessel branch points contained in a circular region described by the filter disk.

### Western Blot analysis

Western Blot experiments were performed as previously described^23^. Briefly, the following primary antibodies were used: rabbit anti-phosho-Akt (Ser473, D9E, Cell Signaling), mouse anti-phospho-p44/42 MAPK (ERK1/2) (Thr202/Tyr204, E10, Cell Signaling), rabbit anti-Phospho-p70 S6 Kinase (Thr389, Cell Signaling) and rabbit anti-β-tubulin (Santa Cruz Biotechnology, USA) antibodies.

### Statistical analysis

The results of all experiments are expressed as mean ±SD. Statistical analyses were performed using, t-test or ANOVA test to compare more than two experimental conditions. A p value below 0.05 was considered significant.

## Author Contributions

Conception and design: F.M., S.O.B., F.V. and E.G. Development of methodology: F.M., S.O.B. and C.M. Data acquisition: F.M., S.O.B. and C.M. Data analysis and interpretation: F.M., S.O.B., C.M., F.D.N., G.B., F.V. and E.G. Writing, review and/or revision of the manuscript: F.M., S.O.B., C.M., F.D.N., F.B., G.B., F.V. and E.G. Study supervision: F.V. and E.G.

## Supplementary Material

Supplementary Information Supplemental Informations

## References

[b1] Folkman J. Role of angiogenesis in tumor growth and metastasis. Seminars in Oncology 29, 15–18 (2002). 12516034 10.1053/sonc.2002.37263

[b2] Paez-Ribes M. *et al.* Antiangiogenic therapy elicits malignant progression of tumors to increased local invasion and distant metastasis. Cancer Cell 15, 220–231 (2009). 19249680 10.1016/j.ccr.2009.01.027 PMC2874829

[b3] Sennino B. & McDonald D. M. Controlling escape from angiogenesis inhibitors. Nat Rev Cancer 12, 699–709 (2012). 23001349 10.1038/nrc3366 PMC3969886

[b4] De Bock K. *et al.* Role of PFKFB3-driven glycolysis in vessel sprouting. Cell 154, 651–663 (2013). 23911327 10.1016/j.cell.2013.06.037

[b5] Schoors S. *et al.* Incomplete and transitory decrease of glycolysis: a new paradigm for anti-angiogenic therapy? Cell Cycle 13, 16–22 (2014). 24335389 10.4161/cc.27519 PMC3925729

[b6] Gorin A., Gabitova L. & Astsaturov I. Regulation of cholesterol biosynthesis and cancer signaling. Curr Opin Pharmacol. 12, 710–716 (2012). 22824431 10.1016/j.coph.2012.06.011 PMC3504641

[b7] Osmak M. Statins and cancer: current and future prospects. Cancer Lett. 324, 1–12 (2012). 22542807 10.1016/j.canlet.2012.04.011

[b8] Zeichner S., Mihos C. G. & Santana O. The pleiotropic effects and therapeutic potential of the hydroxy-methyl-glutaryl-CoA reductase inhibitors in malignancies: a comprehensive review. J Cancer Res Ther. 8, 176–183 (2012). 22842358 10.4103/0973-1482.98967

[b9] Chong C. R. *et al.* Inhibition of angiogenesis by the antifungal drug itraconazole. Acs Chemical Biology 2, 263–270 (2007). 17432820 10.1021/cb600362d

[b10] Ho P. Y. *et al.* Inhibition of human vascular endothelial cells proliferation by terbinafine. International Journal of Cancer 111, 51–59 (2004). 10.1002/ijc.20039 15185342

[b11] Aftab B. T., Dobromilskaya I., Liu J. O. & Rudin C. M. Itraconazole inhibits angiogenesis and tumor growth in non-small cell lung cancer. Cancer Res. 71, 6764–6772 (2011). 21896639 10.1158/0008-5472.CAN-11-0691 PMC3206167

[b12] Kim J. *et al.* Itraconazole, a commonly used antifungal that inhibits Hedgehog pathway activity and cancer growth. Cancer Cell 17, 388–399 (2010). 20385363 10.1016/j.ccr.2010.02.027 PMC4039177

[b13] Chien M. H. *et al.* Terbinafine inhibits oral squamous cell carcinoma growth through anti-cancer cell proliferation and anti-angiogenesis. Mol Carcinog. 51, 389–399 (2012). 21563217 10.1002/mc.20800

[b14] Belter A. *et al.* Squalene monooxygenase - a target for hypercholesterolemic therapy. Biol Chem. 392, 1053–1075 (2011). 22050222 10.1515/BC.2011.195

[b15] Lass-Florl C. Triazole antifungal agents in invasive fungal infections: a comparative review. Drugs 71, 2405–2419 (2011). 22141384 10.2165/11596540-000000000-00000

[b16] Morand O. H. *et al.* Ro 48-8071, a new 2,3-oxidosqualene:lanosterol cyclase inhibitor lowering plasma cholesterol in hamsters, squirrel monkeys, and minipigs: Comparison to simvastatin. Journal of Lipid Research 38, 373–390 (1997). 9162756

[b17] Charlton-Menys V. & Durrington P. N. Squalene synthase inhibitors: clinical pharmacology and cholesterol-lowering potential. Drugs 67, 11–16 (2007). 17209661 10.2165/00003495-200767010-00002

[b18] Bergers G. *et al.* Benefits of targeting both pericytes and endothelial cells in the tumor vasculature with kinase inhibitors. J Clin Invest. 111, 1287–1295 (2003). 12727920 10.1172/JCI17929 PMC154450

[b19] Bergers G. *et al.* Effects of angiogenesis inhibitors on multistage carcinogenesis in mice. Science 284, 808–812 (1999). 10221914 10.1126/science.284.5415.808

[b20] Chuang J. C. *et al.* Sustained and selective suppression of intestinal cholesterol synthesis by Ro 48-8071, an inhibitor of 2,3-oxidosqualene:lanosterol cyclase, in the BALB/c mouse. Biochem Pharmacol. 88, 351–363 (2014). 24486573 10.1016/j.bcp.2014.01.031 PMC3989900

[b21] Goel S. *et al.* Normalization of the vasculature for treatment of cancer and other diseases. Physiol Rev. 91, 1071–1121 (2011). 21742796 10.1152/physrev.00038.2010 PMC3258432

[b22] Serini G., Bussolino F., Maione F. & Giraudo E. Class 3 semaphorins: physiological vascular normalizing agents for anti-cancer therapy. J Intern Med. 273, 138–155 (2013). 23198760 10.1111/joim.12017

[b23] Maione F. *et al.* Semaphorin 3A overcomes cancer hypoxia and metastatic dissemination induced by antiangiogenic treatment in mice. J Clin Invest. 122, 1832–1848 (2012). 22484816 10.1172/JCI58976 PMC3336974

[b24] Adjei A. A. *et al.* Comparative pharmacokinetic study of continuous venous infusion fluorouracil and oral fluorouracil with eniluracil in patients with advanced solid tumors. J Clin Oncol. 20, 1683–1691 (2002). 11896120 10.1200/JCO.2002.20.6.1683

[b25] Chung A. S. & Ferrara N. Developmental and pathological angiogenesis. Annu Rev Cell Dev Biol. 27, 563–584 (2011). 21756109 10.1146/annurev-cellbio-092910-154002

[b26] Del Carratore R. *et al.* Itraconazole inhibits HMEC-1 angiogenesis. Biomed Pharmacother. 66, 312–317 (2012). 22564244 10.1016/j.biopha.2011.11.004

[b27] D'Amore P. A. & Bryan B. A. What tangled webs they weave: Rho-GTPase control of angiogenesis. Cellular and Molecular Life Sciences 64, 2053–2065 (2007). 17530172 10.1007/s00018-007-7008-z PMC11138424

[b28] Cascone I. *et al.* Temporal and spatial modulation of Rho GTPases during in vitro formation of capillary vascular network. Adherens junctions and myosin light chain as targets of Rac1 and RhoA. J Biol Chem. 278, 50702–50713 (2003). 12972426 10.1074/jbc.M307234200

[b29] Jiang B. H. & Liu L. Z. PI3K/PTEN signaling in angiogenesis and tumorigenesis. Adv Cancer Res. 102, 19–65 (2009). 19595306 10.1016/S0065-230X(09)02002-8 PMC2933405

[b30] Graupera M. & Potente M. Regulation of angiogenesis by PI3K signaling networks. Exp Cell Res. 319, 1348–1355 (2013). 23500680 10.1016/j.yexcr.2013.02.021

[b31] Nacev B. A. *et al.* The antifungal drug itraconazole inhibits vascular endothelial growth factor receptor 2 (VEGFR2) glycosylation, trafficking, and signaling in endothelial cells. J Biol Chem. 286, 44045–44056 (2011). 22025615 10.1074/jbc.M111.278754 PMC3243534

[b32] Xu J., Dang Y., Ren Y. R. & Liu J. O. Cholesterol trafficking is required for mTOR activation in endothelial cells. Proc Natl Acad Sci U S A 107, 4764–4769 (2010). 20176935 10.1073/pnas.0910872107 PMC2842052

[b33] Mejia-Pous C., Damiola F. & Gandrillon O. Cholesterol synthesis-related enzyme oxidosqualene cyclase is required to maintain self-renewal in primary erythroid progenitors. Cell Prolif. 44, 441–452 (2011). 21951287 10.1111/j.1365-2184.2011.00771.x PMC6495882

[b34] Grinter S. Z. *et al.* An inverse docking approach for identifying new potential anti-cancer targets. J Mol Graph Model 29, 795–799 (2011). 21315634 10.1016/j.jmgm.2011.01.002 PMC3068237

[b35] Staedler D. *et al.* Cytotoxic effects of combination of oxidosqualene cyclase inhibitors with atorvastatin in human cancer cells. J Med Chem. 55, 4990–5002 (2012). 22533316 10.1021/jm300256z

[b36] Liang Y. *et al.* Cholesterol biosynthesis inhibitors as potent novel anti-cancer agents: suppression of hormone-dependent breast cancer by the oxidosqualene cyclase inhibitor RO 48-8071. Breast Cancer Res Treat. 146, 51–62 (2014). 24878988 10.1007/s10549-014-2996-5 PMC11121502

[b37] Carmeliet P. & Jain R. K. Molecular mechanisms and clinical applications of angiogenesis. Nature 473, 298–307 (2011). 21593862 10.1038/nature10144 PMC4049445

[b38] Pennacchietti S. *et al.* Hypoxia promotes invasive growth by transcriptional activation of the met protooncogene. Cancer Cell 3, 347–361 (2003). 12726861 10.1016/s1535-6108(03)00085-0

[b39] Semenza G. L. Hypoxia and cancer. Cancer Metastasis Rev. 26, 223–224 (2007). 17404692 10.1007/s10555-007-9058-y

[b40] Jain R. K. Antiangiogenesis strategies revisited: from starving tumors to alleviating hypoxia. Cancer Cell 26, 605–622 (2014). 25517747 10.1016/j.ccell.2014.10.006 PMC4269830

[b41] Rapisarda A. & Melillo G. Overcoming disappointing results with antiangiogenic therapy by targeting hypoxia. Nat Rev Clin Oncol. 9, 378–390 (2012). 22525710 10.1038/nrclinonc.2012.64

[b42] Matters G. L. *et al.* Cholecystokinin mediates progression and metastasis of pancreatic cancer associated with dietary fat. Dig Dis Sci. 59, 1180–1191 (2014). 24817409 10.1007/s10620-014-3201-8 PMC4096234

[b43] Brown S. R., Hossain M. B. & Forrester I. T. Associations between cholesterol, colon cancer screening, behavior, and diet. Am J Health Behav. 37, 360–368 (2013). 23985183 10.5993/AJHB.37.3.9

[b44] Carey F. J. *et al.* The differential effects of statins on the risk of developing pancreatic cancer: a case-control study in two centres in the United Kingdom. Dig Dis Sci. 58, 3308–3312 (2013). 23864194 10.1007/s10620-013-2778-7

[b45] Fang Z. *et al.* Simvastatin inhibits renal cancer cell growth and metastasis via AKT/mTOR, ERK and JAK2/STAT3 pathway. PLoS One 8, e62823 (2013). 23690956 10.1371/journal.pone.0062823 PMC3656850

[b46] Sarbassov D. D., Ali S. M. & Sabatini D. M. Growing roles for the mTOR pathway. Curr Opin Cell Biol. 17, 596–603 (2005). 16226444 10.1016/j.ceb.2005.09.009

[b47] Sheppard K. *et al.* Targeting PI3 kinase/AKT/mTOR signaling in cancer. Crit Rev Oncog. 17, 69–95 (2012). 22471665 10.1615/critrevoncog.v17.i1.60

[b48] Riobo N. A. Cholesterol and its derivatives in Sonic Hedgehog signaling and cancer. Curr Opin Pharmacol. 12, 736–741 (2012). 22832232 10.1016/j.coph.2012.07.002 PMC3675894

[b49] Kanda S. *et al.* Sonic hedgehog induces capillary morphogenesis by endothelial cells through phosphoinositide 3-kinase. J Biol Chem. 278, 8244–8249 (2003). 12514186 10.1074/jbc.M210635200

[b50] Fu J. R. *et al.* Sonic hedgehog protein promotes bone marrow-derived endothelial progenitor cell proliferation, migration and VEGF production via PI 3-kinase/Akt signaling pathways. Acta Pharmacol Sin. 27, 685–693 (2006). 16723086 10.1111/j.1745-7254.2006.00335.x

[b51] Lauth M. & Toftgard R. Hedgehog signaling and pancreatic tumor development. Adv Cancer Res. 110, 1–17 (2011). 21704226 10.1016/B978-0-12-386469-7.00001-3

[b52] Xu M. *et al.* Prognostic value of hedgehog signaling pathway in patients with colon cancer. Med Oncol. 29, 1010–1016 (2012). 21424326 10.1007/s12032-011-9899-7

[b53] Bussolino F. *et al.* Hepatocyte growth factor is a potent angiogenic factor which stimulates endothelial cell motility and growth. The Journal of Cell Biology 119, 629–641 (1992). 1383237 10.1083/jcb.119.3.629 PMC2289675

[b54] Fujisawa T., Joshi B., Nakajima A. & Puri R. K. A novel role of interleukin-13 receptor alpha2 in pancreatic cancer invasion and metastasis. Cancer Res. 69, 8678–8685 (2009). 19887609 10.1158/0008-5472.CAN-09-2100

[b55] McIntyre A. *et al.* Carbonic anhydrase IX promotes tumor growth and necrosis in vivo and inhibition enhances anti-VEGF therapy. Clin Cancer Res. 18, 3100–3111 (2012). 22498007 10.1158/1078-0432.CCR-11-1877 PMC3367109

[b56] Casazza A. *et al.* Systemic and targeted delivery of semaphorin 3A inhibits tumor angiogenesis and progression in mouse tumor models. Arterioscler Thromb Vasc Biol. 31, 741–749 (2011). 21205984 10.1161/ATVBAHA.110.211920

[b57] Chou T. C. Drug combination studies and their synergy quantification using the Chou-Talalay method. Cancer Res. 70, 440–446 (2010). 20068163 10.1158/0008-5472.CAN-09-1947

[b58] Valdembri D. *et al.* Neuropilin-1/GIPC1 signaling regulates alpha5beta1 integrin traffic and function in endothelial cells. PLoS Biol. 7, e25 (2009). 19175293 10.1371/journal.pbio.1000025 PMC2631072

[b59] Maione F. *et al.* Semaphorin 3A is an endogenous angiogenesis inhibitor that blocks tumor growth and normalizes tumor vasculature in transgenic mouse models. J Clin Invest. 119, 3356–3372 (2009). 19809158 10.1172/JCI36308 PMC2769187

[b60] Naldini A. *et al.* Cutting edge: IL-1 beta mediates the proangiogenic activity of osteopontin-activated human monocytes. Journal of Immunology 177, 4267–4270 (2006). 10.4049/jimmunol.177.7.4267 16982859

