# Supplementary material for: The cholesterol biosynthesis enzyme oxidosqualene cyclase is a new target to impair tumour angiogenesis and metastasis dissemination
Source: Sci Rep. 2015 Mar 12;5:9054. doi: 10.1038/srep09054 (PMC4357009; doi:10.1038/srep09054)

The cholesterol biosynthesis enzyme oxidosqualene cyclase is a new target to impair tumor angiogenesis and metastasis dissemination

Federica Maione, Simonetta Oliaro-Bosso, Claudia Meda, Federica Di Nicolantonio, Federico Bussolino, Gianni Balliano, Franca Viola and Enrico Giraudo

SUPPLEMENTARY FIGURE LEGENDS

Supplementary Fig. S1. **Histological analysis of healthy organs from mice treated with Ro 48-8071.** (A) Graph shows the relative body weight of a cohort of RIP-Tag2 mice during Ro 48-8071 treatment. No variations due to a possible side effect of the drug were found in mice during all the treatment. (B-D) Histological examination of healthy organs from control animals and mice treated with Ro 48-8071. Paraffin-embedded organs were sectioned and stained with hematoxylin/eosin (H&E). At the doses tested no toxicity of Ro 48-8071 was observed by H&E analysis of kidney (B), lung (C) or liver (D) taken at necropsy. Their histology was comparable between the mice treated with Ro 48-8071 and controls.

Supplementary Fig. S2. **Ro 48-8071 treatment did not affect normal vessels of exocrine pancreas.** A) Fresh frozen tissues from control and Ro 48-8071 treated RIP-Tag2 mice were stained with Meca32 (BD Pharmingen) antibody. Exocrine normal pancreas surrounding tumor islets was evaluated by confocal microscopy analysis. B) Vessel density was obtained from the quantification of at least five ×400 power field/ animal pictures by means of the Leica Confocal Software Histogram Quantification Tool. Scale bars: 50 μm.

Supplementary Fig. S3. **Effect of Ro 48-8071 on tumor hypoxia index in RIP-Tag2 mice, HCT116 and HPAF-II mouse models** The amount of tumor hypoxia was determined 2 hours after injection of 60 mg/kg pimonidazole hydrochloride (HP2- 100 Hypoxyprobe Kit-Plus; Natural Pharmacia International Inc.) into HCT116 (A), HPAF-II (B) and RIP-Tag2 (C) mice. The formation of pimonidazole adducts was detected by immunostaining with Hypoxyprobe-1-Mab1 FITC Ab according to the manufacturer's instructions. D) Quantification was done by means of Image-ProPlus 6.2 software (Media Cybernetics), analyzing at least 5 sections and 5 fields per tumor.

Supplementary Fig. S4. Effect of Ro 48-8071 on the proliferation index in RIP-Tag2 mice, HCT116 and HPAF-II mouse models. Proliferation rate was assessed by immunostaining using Ki-67 antibody on frozen tissues from 8 animals/group. In RIP-Tag2 (A) HCT116 (B) and HPAF-II (C) xenografts a decrease of cell proliferation was observed. Images are representative of 10 fields per mouse. Optical images of Ki-67 immunostainings were visualized with a BX-60 microscope (Olympus) equipped with a color Qicam Fast 1394-digital CCD camera (12 bit; QImaging). Proliferation rate was quantified by counting in a 10X field the number of Ki-67 positive/total cells by means of ImageJ software.

Supplementary Fig. S5. Effect of Ro 48-8071 on cell proliferation in HCT116 and HPAF-II tumor cell lines and HUVEC. The cell proliferation was evaluated by determining cell density by the sulforhodamine B colorimetric assay (SRB assay) at 24 and 72 h after seeding 3,000 cells into 96-well plates in complete medium in presence of increasing concentrations of Ro 48-8071 (0.1, 1 10, 30 μM). SRB assay, was performed as previously described [1]. Ro 48-8071 did not exert a direct effect on HCT116 (A), HPAF-II (B) HUVEC (C) and cell growth. Cell proliferation is indicated as (T/C%) (mean OD of treated cells/mean OD of control cells x 100). Values are mean ± SD (n=3 wells/condition) of 2 independent experiments.

Supplementary Fig. S6. Matrigel invasion assays in HCT116 and HPAF-II tumor cell lines. This assay was performed in trans-well chambers (BD). The upper side of the porous polycarbonate membrane (8.0 μm pore size) was coated with 20 μg/well of Matrigel (Becton Dickinson). HCT116 (A) or HPAF-II (B) (2.5×10^5/well) were seeded on the upper side of the filter and incubated in medium+0.5% FCS. The lower chamber was filled with medium containing 1μM Ro 48-8071 or 0.5% FCS as control. After 16h, cells on the upper side of the filters were mechanically removed, while cells migrated to the lower side were fixed and stained with crystal violet. C) The filters were photographed and cells were semi-quantitatively counted by means of ImageJ software.

Supplementary Fig. S7. Ro 48-8071 inhibits vessel formation in Matrigel and CAM assays more efficiently than itraconazole. (A) EC morphogenesis was evaluated by Matrigel assay. The tubular vessel network formation was significantly impaired by 1μM Ro 48-8071 and 1μM

itraconazole (Amersham Pharmacia Biotech, UK) compared with controls. Of note, Ro 48-8071 was more efficient (by 47%) in inhibiting vessel formation compared with itraconazole. Values are mean ± SD (*$p<0.05$, ***$p<0.001$; ANOVA test). ECs tube length was measured by the software winRHIZO Pro (Regent Instruments Inc). Images are representative of 3 independent experiments. (B) CAM assay. Bar graph shows the number of vessels per implant. FGF-2-induced vessel formation was strongly impaired by 1μM Ro 48-8071 and 1μM itraconazole, compared with controls. Remarkably, as for the Matrigel assay, Ro 48-8071 more strongly impaired FGF-2-induced vessel formation (by 58%) compared with itraconazole. Data are presented as mean ± SD of 18 embryos per treatment (**$p<0.01$***$p<0.001$, ANOVA test).

Supplementary Fig. S8. The inhibition of OSC does not interfere with RhoA activation in ECs. (**A**) The active Rho pull-down and detection kit (Thermo Scientific) was used according to the manufacturer's instructions. Western blotting analysis of active RhoA pull-down from lysates of ECs treated with Ro 48-8071 for 24 hours. Relative active RhoA GTP-bound level was normalized on total RhoA protein amount. (**B**) Bar graph shows the quantification of active RhoA-GTP levels on total RhoA and it is representative of 5 independent experiments. Values are mean ± SD.

Supplementary Fig. S1

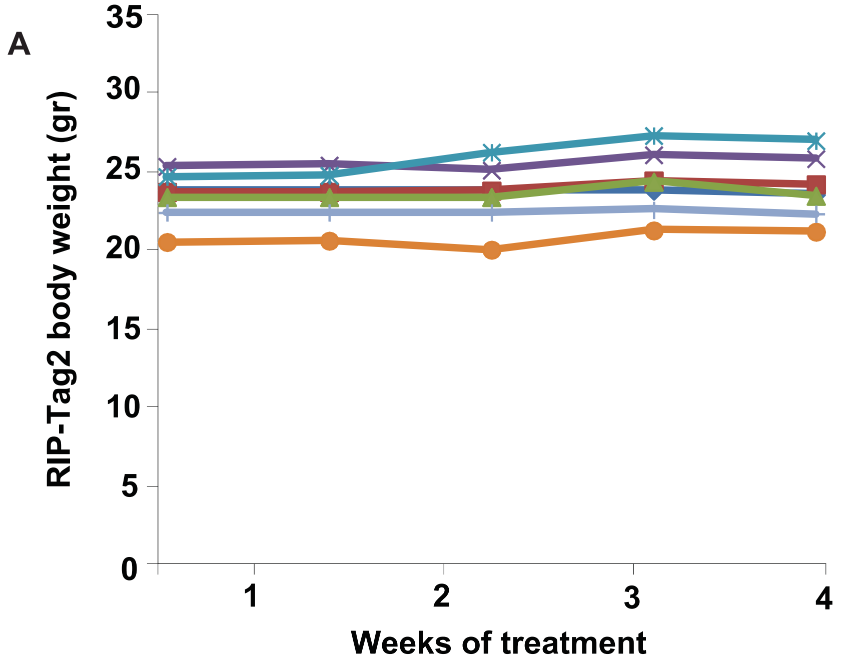

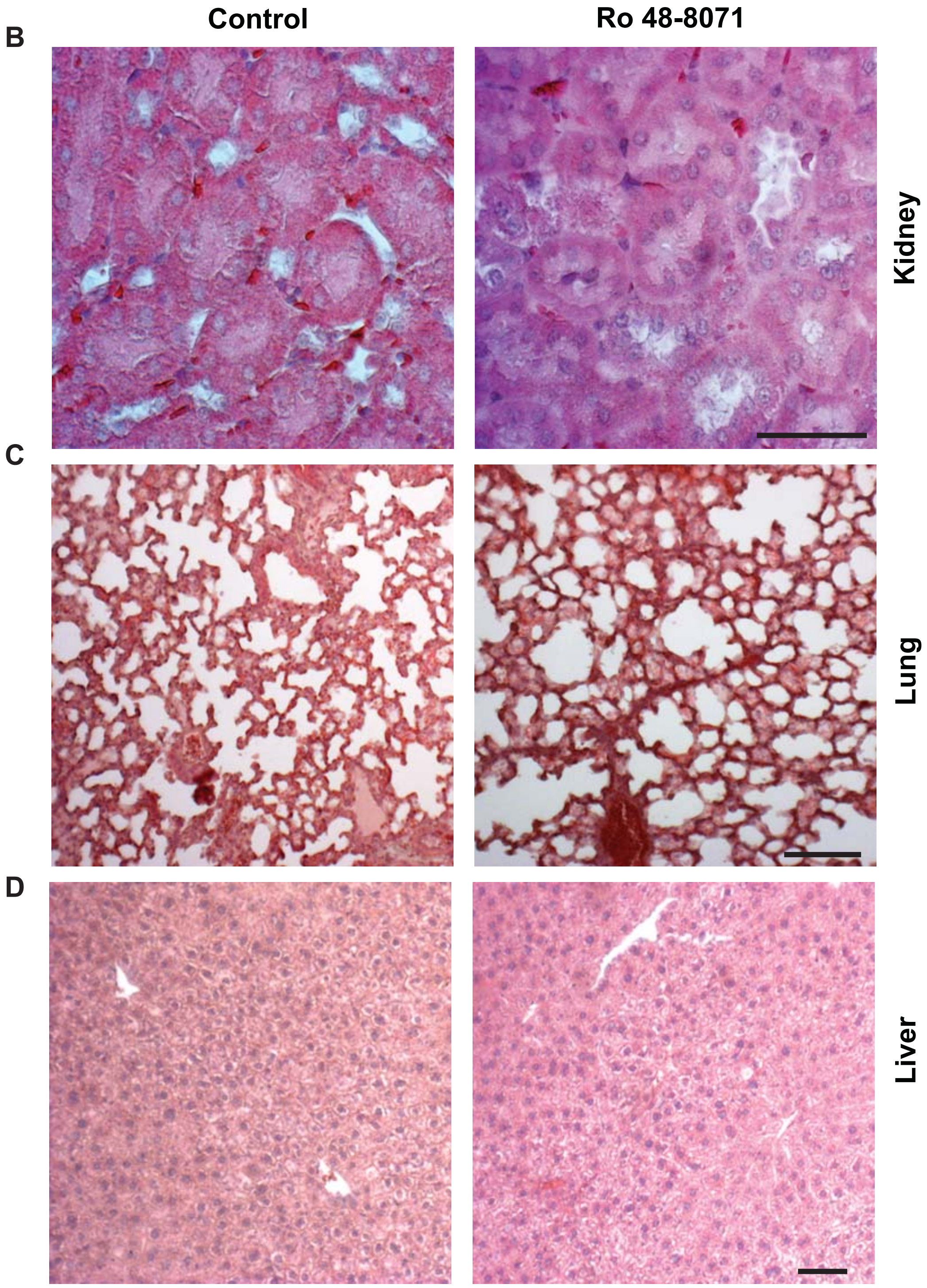

Supplementary Fig. S2

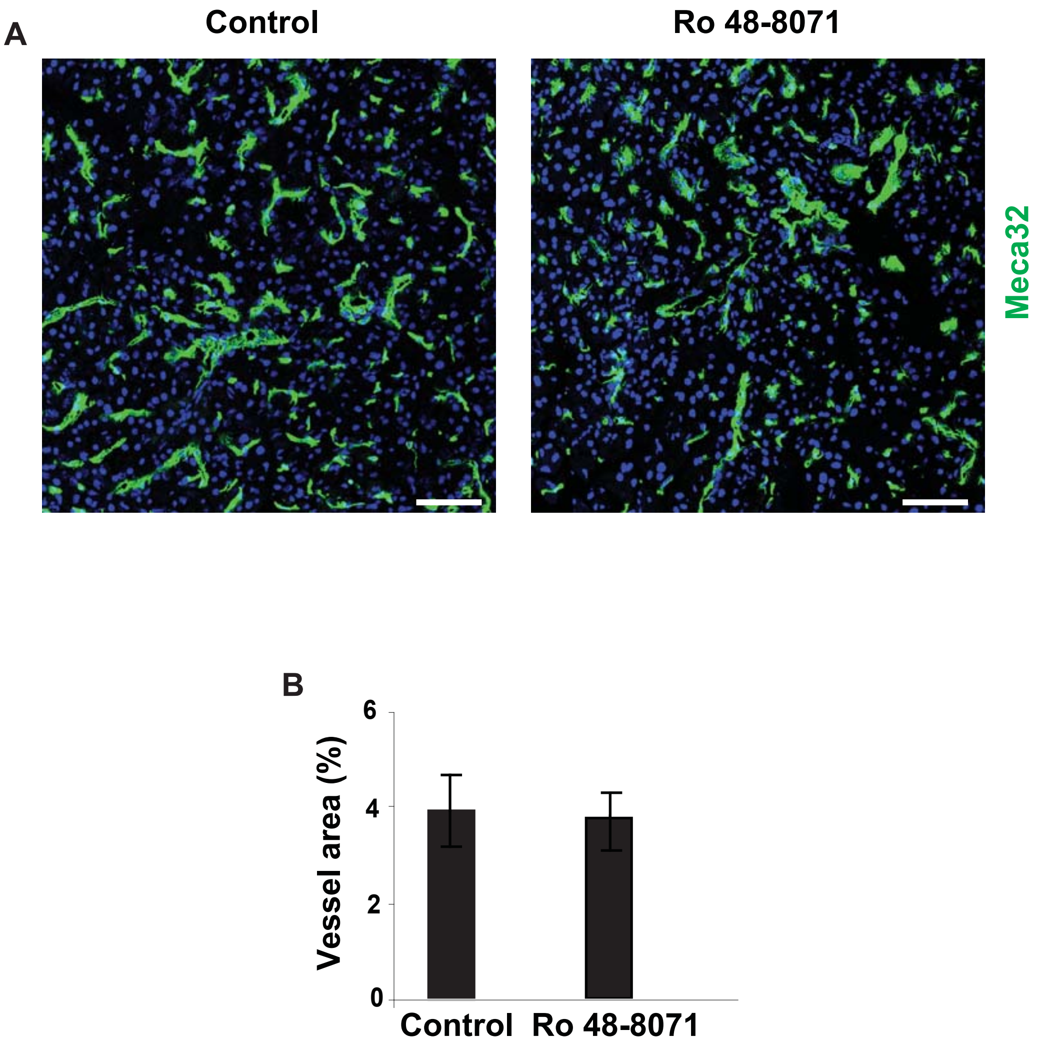

Supplementary Fig. S3

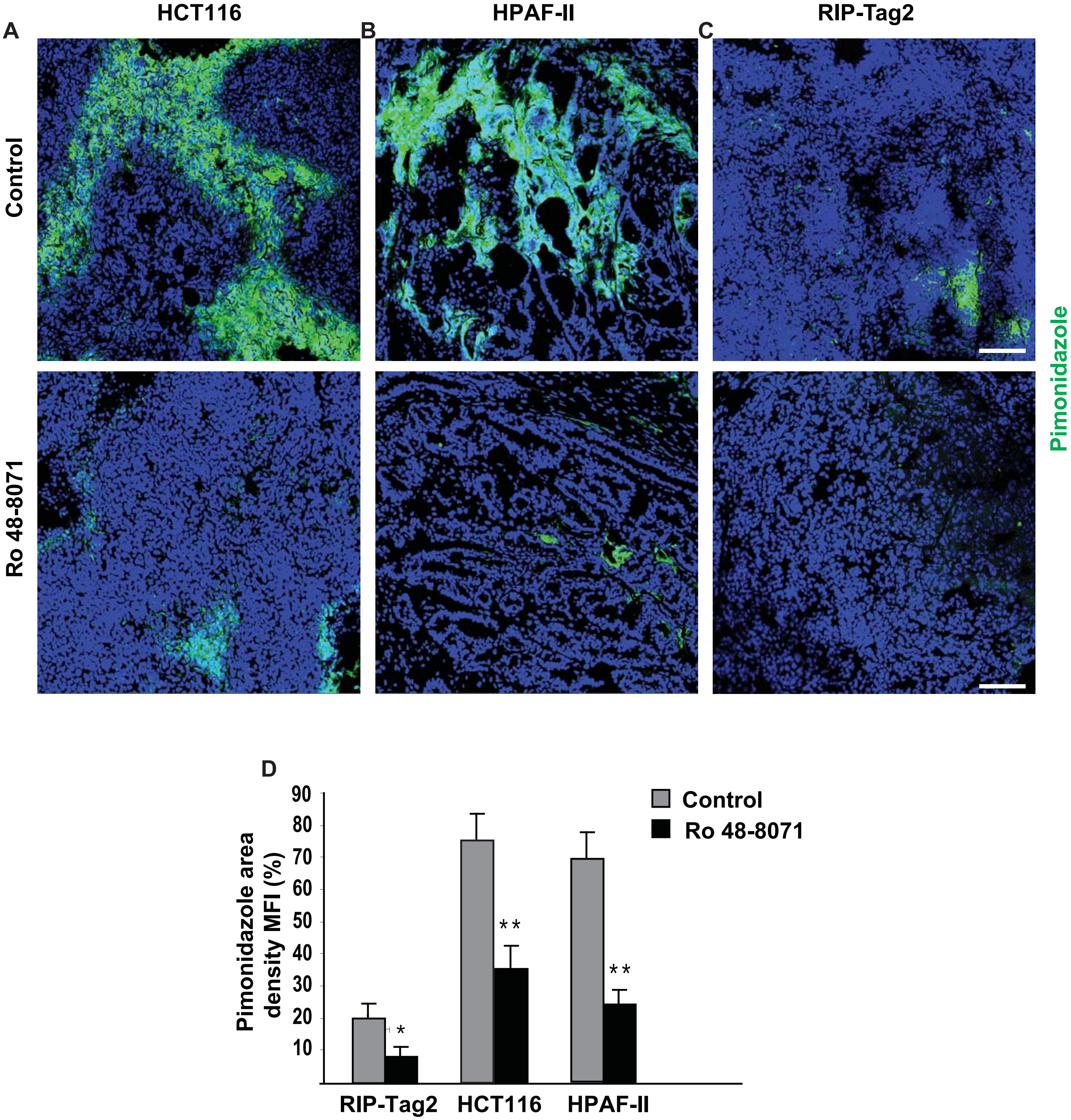

Supplementary Fig. S4

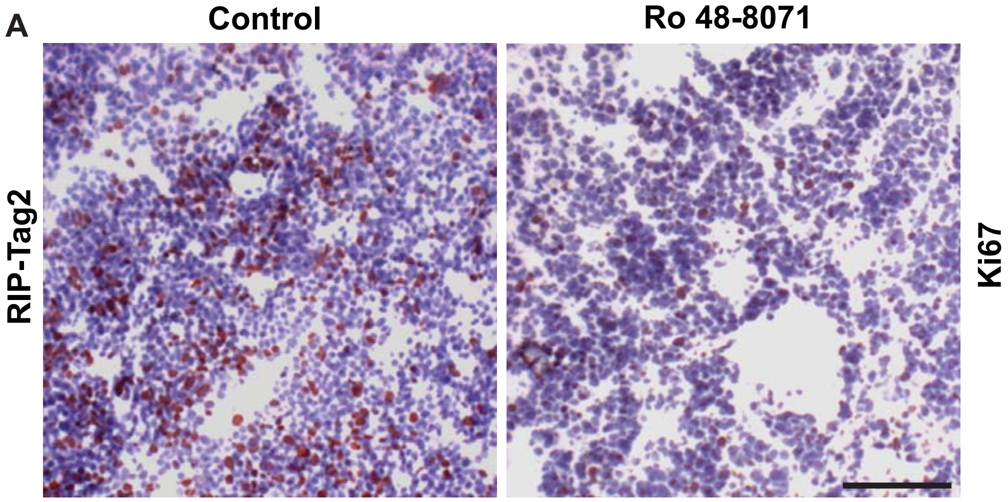

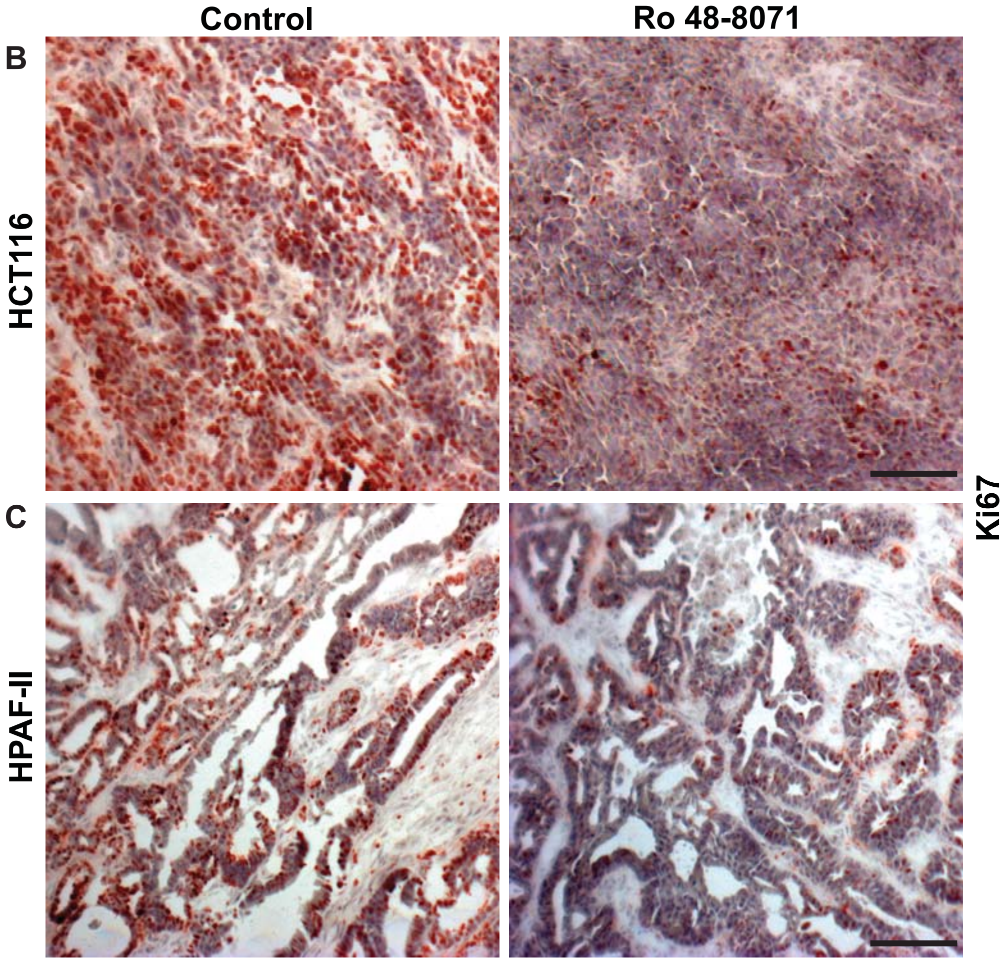

Supplementary Fig. S5

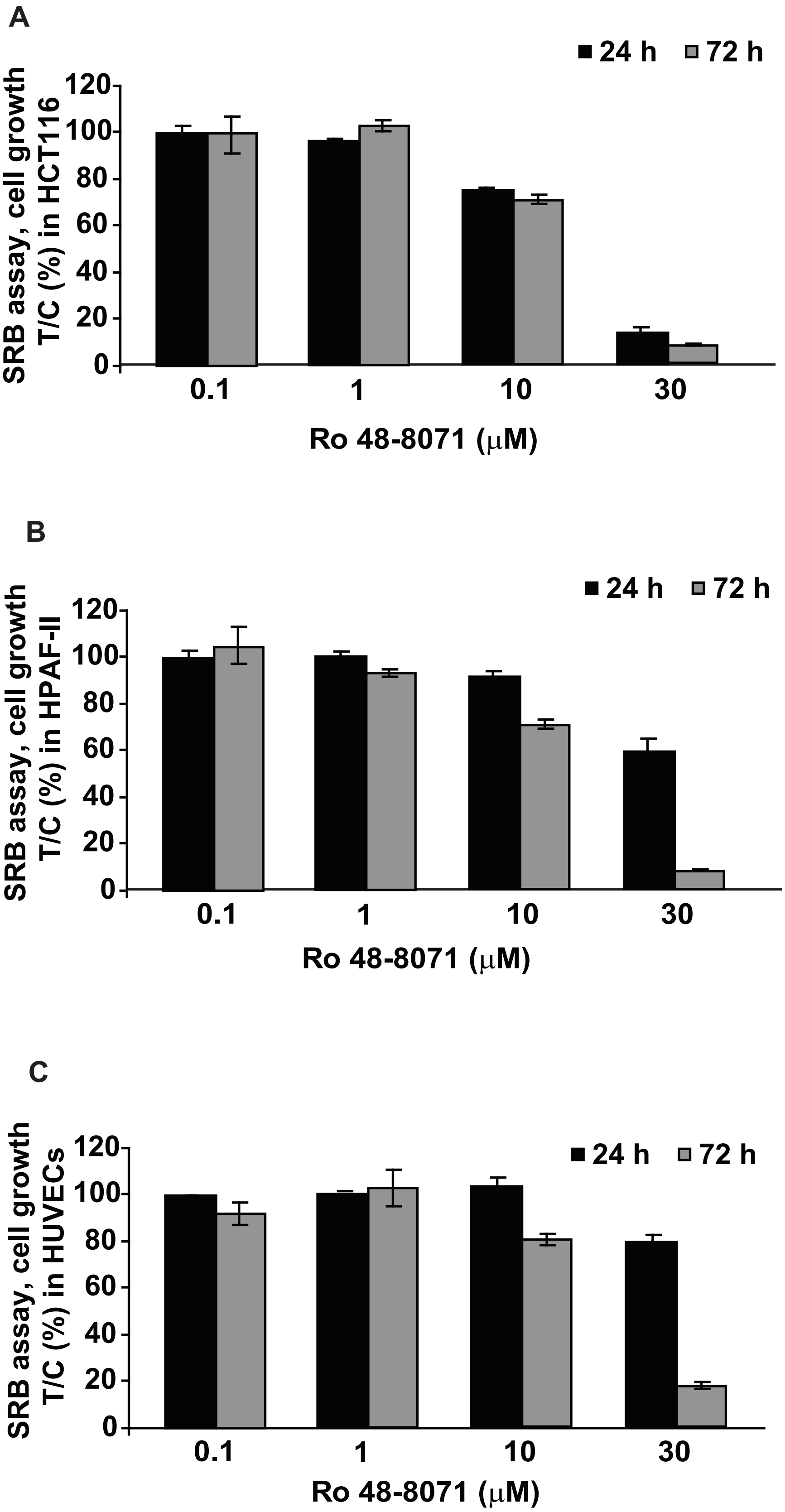

Supplementary Fig. S6

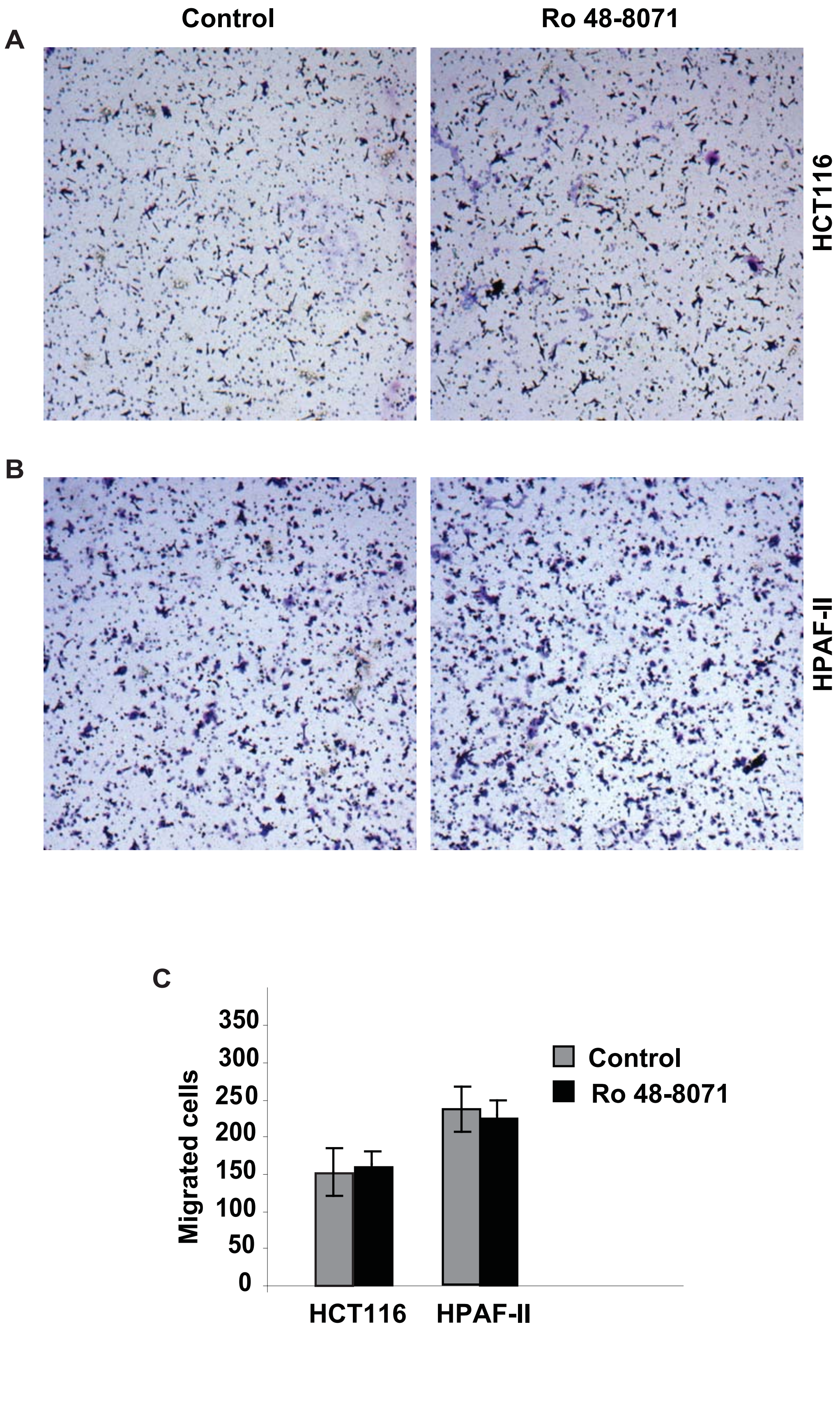

Supplementary Fig. S7

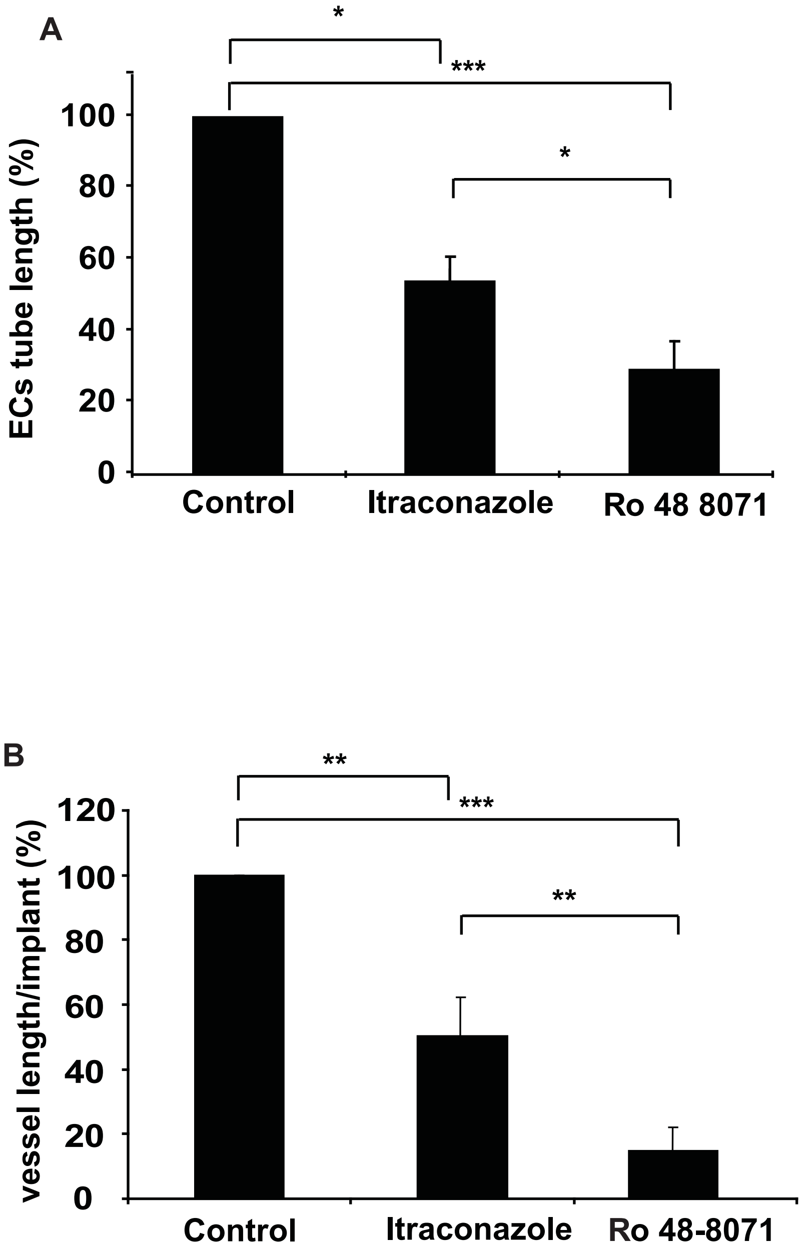

Supplementary Fig. S8

A

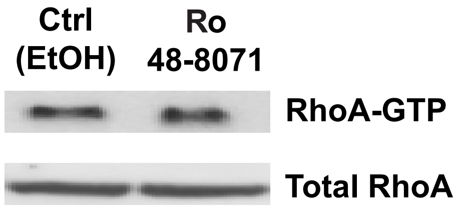

B

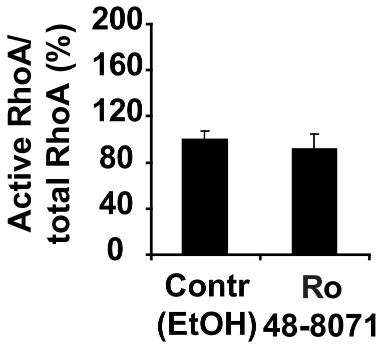

Supplement: Supplementary Information — Supplemental Informations [file srep09054-s1.pdf]
